# The white matter is a pro-differentiative niche for glioblastoma

**DOI:** 10.1038/s41467-021-22225-w

**Published:** 2021-04-12

**Authors:** Lucy J. Brooks, Melanie P. Clements, Jemima J. Burden, Daniela Kocher, Luca Richards, Sara Castro Devesa, Leila Zakka, Megan Woodberry, Michael Ellis, Zane Jaunmuktane, Sebastian Brandner, Gillian Morrison, Steven M. Pollard, Peter B. Dirks, Samuel Marguerat, Simona Parrinello

**Affiliations:** 1 Samantha Dickson Brain Cancer Unit, UCL Cancer Institute, London, WC1E 6DD UK; 2 MRC Laboratory for Molecular Cell Biology, University College London, Gower Street, London, WC1E 6BT UK; 3 Division of Neuropathology, National Hospital for Neurology and Neurosurgery, University College London NHS Foundation Trust, Queen Square, WC1N 3BG London, UK; 4 Department of Neurodegenerative Disease, UCL Institute of Neurology, Queen Square, WC1N 3BG London, UK; 5 MRC Centre for Regenerative Medicine and Edinburgh Cancer Research UK Cancer Centre, University of Edinburgh, 5 Little France Drive, Edinburgh, EH16 4UU UK; 6 Division of Neurosurgery, Arthur and Sonia Labatt Brain Tumor Research Center, Departments of Surgery and Molecular Genetics, Hospital for Sick Children, Toronto, ON M5G 1X8 Canada; 7 MRC London Institute of Medical Sciences, Du Cane Road, London, W12 0NN UK; 8 Institute of Clinical Sciences, Faculty of Medicine, Imperial College London, Du Cane Road, London, W12 0NN UK

**Keywords:** Cancer microenvironment, Cancer stem cells, CNS cancer

## Abstract

Glioblastomas are hierarchically organised tumours driven by glioma stem cells that retain partial differentiation potential. Glioma stem cells are maintained in specialised microenvironments, but whether, or how, they undergo lineage progression outside of these niches remains unclear. Here we identify the white matter as a differentiative niche for glioblastomas with oligodendrocyte lineage competency. Tumour cells in contact with white matter acquire pre-oligodendrocyte fate, resulting in decreased proliferation and invasion. Differentiation is a response to white matter injury, which is caused by tumour infiltration itself in a tumoursuppressive feedback loop. Mechanistically, tumour cell differentiation is driven by selective white matter upregulation of SOX10, a master regulator of normal oligodendrogenesis. SOX10 overexpression or treatment with myelination-promoting agents that upregulate endogenous SOX10, mimic this response, leading to niche-independent pre-oligodendrocyte differentiation and tumour suppression in vivo. Thus, glioblastoma recapitulates an injury response and exploiting this latent programme may offer treatment opportunities for a subset of patients.

## Abstract (web summary)

Glioma stem cells (GSCs) retain the ability to partially differentiate, but it is unclear how the brain microenvironment may influence this response. Here the authors show that glioblastoma cells infiltrating into the white matter acquire pre-oligodendrocyte-like fate in a process that mimics myelin repair and results in tumour suppression

## Introduction

Glioblastoma (GBM) is the most common and malignant primary brain tumour^1^. Stark resistance to current treatments, which include maximal surgical resection, chemo- and radiotherapy, leads to tumour relapse in virtually all patients with a median survival of less than 15 months^2^. GBM initiation, growth and recurrence are thought to be rooted within a subpopulation of therapy-resistant tumour cells with properties of normal neural stem cells, termed glioma stem cells (GSCs)^3–7^.

Mounting evidence indicates that GSCs fuel tumourigenesis by recapitulating normal neural lineage hierarchies of quiescence, self-renewal and generation of non-dividing progeny^8–10^. Fate decisions of normal neural stem cells are tightly controlled by the microenvironment, which maintains stemness in specialised niches and directs differentiation along the appropriate lineages^11^. Similarly, it is well established that GSCs are maintained in perivascular and hypoxic regions of the tumour bulk^12–14^. In contrast, little is known about the differentiation potential of GSCs within tumours and whether pro-differentiative niches exist. However, improved understanding of lineage progression is central to our understanding of the disease and may reveal novel strategies to suppress GBM growth and recurrence by directing GSC fate towards a mature, non-dividing state. Consistent with this idea, manipulation of candidate developmental pathways to promote tumour cell differentiation into astrocyte and neuronal lineages has shown efficacy in preclinical models^10,15^.

Diffuse infiltration into the normal brain parenchyma is a hallmark of GBM and underlies recurrence by precluding complete surgical resection^16,17^. As GBM cells infiltrate away from the tumour bulk, they are confronted with new and heterogeneous microenvironments, which would be predicted to affect fate decisions^18^. Indeed, it has been proposed that invading GSCs may lose stemness, but this remains controversial, with studies both in support and against this idea^19–22^. A predominant route of infiltration is the white matter, which consists of bundles of myelinated axons, known as tracts, and represents approximately 60% of the brain^16,23^. GBMs frequently develop within white matter and spread throughout the brain using myelinated fibres as scaffolds for cell migration^24^. This includes spread to the contralateral hemisphere, which occurs exclusively along the white matter tracts of the corpus callosum^16^. Remarkably, the cellular and molecular mechanisms that underpin white matter invasion remain almost entirely unknown.

Here, we investigated the impact of the white matter microenvironment on tumour cell fate by profiling region-specific transcriptomes of GBM cells invaded into white and grey matter, alongside matched bulk cells. Unexpectedly, this analysis revealed that the white matter dominantly suppresses malignancy by directing GSC differentiation towards pre-oligodendrocyte fate. We show that this process recapitulates an injury response and can be harnessed to suppress tumourigenicity in a subset of GBMs.

## Results

### Region-specific GBM transcriptomes

**Fig. 1:**
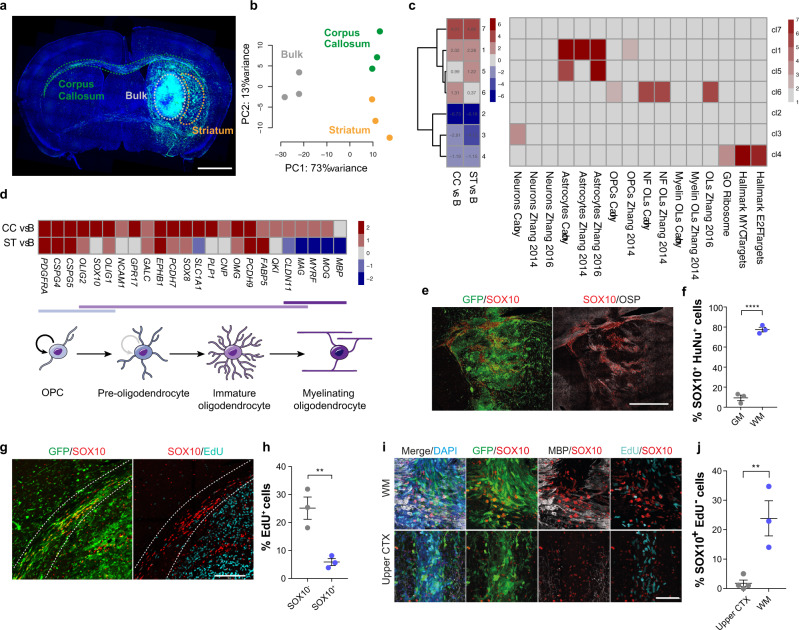
GBM cells invading into white matter acquire pre-oligodendrocyte fate. **a** representative fluorescence image of a G144 xenograft (PDX144) collected for region-specific RNA-seq. Brain regions microdissected for FACS purification of GFP^+^ cells are shown, nuclei are counterstained with DAPI (blue). Scale = 1 mm. **b** PCA plot of rlog-normalised DESeq2 expression scores for the resulting RNA-seq libraries. *n* = 3 xenografts. **c** left panel: K-means clustering of DESeq2 expression ratios (absolute log_2_ expression ratio > 0.58, p_adjust_ < 0.05) for tumour cells invading into the corpus callosum (CC) or striatum (ST) relative to the tumour bulk. One sided Fisher exact test with Bonferroni multiple comparisons test. B Right panel: overlap of K-means cluster with gene signatures for oligodendrocyte progenitor cell (OPC), Oligodendrocytic, Astrocytic and Neuronal lineages as well as proliferation signatures. Colours represent -log_10_ of enrichment adjusted p-values. **d** top: DESeq2 log_2_ expression ratios of markers of the oligodendrocyte lineage organised by differentiation stage in tumour cells from CC and ST relative to B. bottom: schematic illustration of the maturation of oligodendrocyte lineage cells. **e** representative immunofluorescence staining for SOX10 (red) and the myelin marker oligodendrocyte-specific protein (OSP, grey) of GFP-labelled PDX144 tumours (green). Scale = 1 mm. **f** quantification of percentage of SOX10^+^ tumour cells in grey (GM) and white matter (WM). ≥750 cells per xenograft were counted. Mean ± SEM, *n* = 3 xenografts *p* = 0.000047. Unpaired two-tailed Student’s t test. **g** representative EdU (turquoise) and SOX10 (red) staining and **h** quantification of actively proliferating SOX10^+^ and SOX10^−^ GFP^+^ tumour cells in the corpus callosum. Scale = 500 µm. ≥1000 cells per xenograft were counted. Mean ± SEM, *n* = 3 xenografts. *p* = 0.01, unpaired two-tailed Student’s t test. **i** representative SOX10^+^ (red)/EdU^−^ (Turquoise) immunofluorescence staining and **j** quantification of GFP^+^ tumour cells implanted in the white matter of the corpus callosum (top) or the grey matter of the upper cortex (bottom). ≥140 cells per xenograft were counted. Scale = 100 µm, Mean ± SEM, *n* = 3 xenografts *p* = 0.008. Unpaired two-tailed Student’s t test.

Despite the fundamental role of white matter in GBM biology, how this specialised microenvironment might modulate tumour cell behaviour remains unknown. To probe white matter phenotypes, we made use of a well characterised patient-derived GSC line with propensity to invade along white matter tracts (G144)^25^. GFP-labelled G144 cells were stereotactically injected into the striatum of immunocompromised mice and, upon development of clinically apparent disease, the tumour bulk (B) and margin regions extending into the corpus callosum (CC) as a region of white matter, and the striatum (ST) as a region of grey matter, were micro-dissected under fluorescence guidance (Fig. 1a). Tumour tissue was dissociated to single cells and GFP^+^ tumour cells FACS-purified, pooled and processed for RNA-sequencing (RNA-seq). Bioinformatics analysis of human tumour reads revealed that margin cells are transcriptionally distinct from bulk cells, with invasive cells acquiring markers of lineage progression towards astrocytes, as well as downregulating proliferation signatures, which resulted in decreased EdU incorporation in vivo (Fig. 1b, c, Supplementary Fig. 1a–e, Supplementary Table 1 and Supplementary data 1–3)^26–28^. Strikingly, it also indicated that the transcriptomes of CC and ST tumour cells differed from one another (Fig. 1b). Tumour cells invading into white matter selectively upregulated signatures of oligodendroglia, suggesting that myelinated regions may promote progression along the oligodendrocyte lineage (Fig. 1c, Supplementary Fig. 1f and Supplementary Table 1, and Supplementary data 1–3). In agreement with this, CC cells upregulated a panel of oligodendrocyte lineage marker genes, including the transcription factor *SOX10*, a master regulator of oligodendrogenesis (Fig. 1d)^26,29,30^. Notably, upregulation of myelin genes was mild and incomplete in the CC, indicative of partial differentiation, as expected from cancer cells^31^. To determine whether these gene expression changes also correlated with phenotypic changes, we characterised the differentiation response at the single cell level in two sets of complementary experiments. First, we dissociated G144 cells from the CC, ST and B of xenografts, seeded them acutely in mitogen-free media and assessed expression of SOX10, the pre-oligodendrocyte marker O4 and the myelinating oligodendrocyte marker myelin basic protein (MBP) by immunocytochemistry (Supplementary Fig. 1g, h). Preparations isolated from the CC had a strong increase in the proportion of cells positive for SOX10, the majority of which also expressed O4, but not MBP, confirming that transcriptomic signatures reflect changes in tumour cell fate. Second, we examined lineage marker expression within xenografts in situ by immunohistochemistry. GFP or the human-specific nuclear antigen (HuNu) were used to label tumour cells and distinguish them from endogenous mouse glia (Supplementary Fig. 1i, j). The majority of tumour cells began to express high levels of SOX10 in white matter, including in myelinated fibres inside the tumour bulk (Fig. 1e, f). SOX10^+^ tumour cells were significantly less proliferative than tumour cells that remained SOX10^-^ within the same region (Fig. 1g,h) and occasionally expressed the immature oligodendrocyte markers CNP and CC1 (Supplementary Fig. 1k, l), but were again negative for the mature marker MBP (Supplementary Fig. 1m). To further assess the white matter specificity of this response, we implanted G144 cells directly into cortical grey matter or in the corpus callosum and examined their differentiation using SOX10 induction as a read-out. Differentiated SOX10^+^/EdU^−^ tumour cells were only found in the CC, confirming that the white matter selectivey promotes partial GBM differentiation (Fig. 1i,j).

The observed differentiation block of GBM cells is likely due to amplification of PDGFRA in this line, a key negative regulator of developmental oligodendrocyte differentiation which is commonly mutated in proneural/OPC-like GBM cells^9^. Indeed, overexpression of constitutively active PDGFRA in cultured mouse neural stem cells arrested their differentiation at the O4^+^ pre-oligodendrocyte stage, confirming that failure to switch off PDGFRA signalling plays an important role in preventing terminal differentiation of tumour cells (Supplementary Fig. 1n–p). Thus, GBM cells differentiate to a pre-oligodendrocyte/immature pre-oligodendrocyte state in white matter, which is marked by induction of SOX10 expression.

**Fig. 2:**
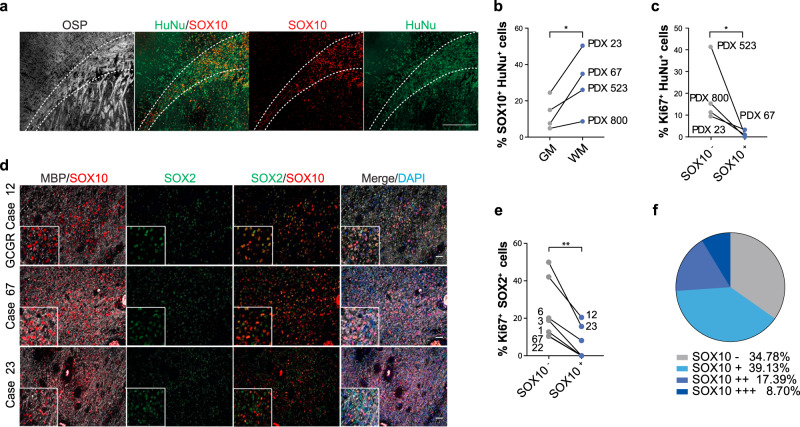
Differentiation in white matter is a general GBM response. **a** representative immunofluorescence image of a SOX10^+^ patient-derived xenograft (PDX 23) stained for SOX10 (red), oligodendrocyte-specific protein (OSP, grey) and human-specific nuclear antigen (HuNu, green) to label all tumour cells. Scale = 500 µm. **b** quantification of percentage of SOX10^+^/HuNu^+^ tumour cells in white and grey matter of indicated patient-derived xenografts. *n* = 4 independent xenograft models. For each xenograft, ≥550 cells were quantified across 2 independent ROIs selected within white or grey matter. *p* = 0.05, Mean, paired one-tailed Student’s t test. **c** quantification of percentage of proliferating SOX10^+^ and SOX10^−^ tumour cells in the corpus callosum of indicated xenografts. For each xenograft, ≥500 SOX10^+^ or SOX10^−^ cells were quantified across 2 independent ROIs, *n* = 4 independent xenograft models. *p* = 0.05, Mean, paired one-tailed Student’s t-test. **d** MBP (grey) and SOX10 (red) immunofluorescence staining of white matter regions of patient tumours. SOX2 (green) was used to identify tumour cells and distinguish them from resident glia. Cases shown are the original tumours from which lines GL23, GL67 and GCGR L12 used in this study have been isolated. Inset shows a close-up of the same image. Scale = 50 µm. **e** quantification of percentage of SOX2^+^/SOX10^+^ and SOX2^+^/SOX10^−^ tumour cells undergoing proliferation (Ki67^+^) in patient tumours. For each case, ≥300 cells were quantified across 2 independent ROIs, *n* = 7 cases. *P* = 0.002, Mean, paired one-tailed Student’s t-test. **f** pie chart representation of SOX10 expression in 23 patient tumours. – denotes absence, and + to +++ increasing frequency of SOX10^+^ cells within the tumour.

To determine the generality of these findings, we examined *SOX10* expression in the TCGA dataset and in a panel of primary GSC lines by qPCR. Consistent with previous studies, we found that a subset of patient tumours (~18%) expressed high levels of *SOX10*. *SOX10* high tumours were enriched for the proneural, but also included mesenchymal and classical transcriptional subtypes, as well as *IDH1* mutant tumours, suggesting that pre-oligodendrocyte differentiation may occur across all GBMs (Supplementary Fig. 2a, b)^9^. In addition, a subset of primary GSC lines retained above average *SOX10* expression in vitro, confirming cell-intrinsic expression in tumour cells (Supplementary Fig. 2c and Supplementary Table 2). We next xenotransplanted five SOX10^+^ GSC lines (three of which were freshly dissociated from primary tumours) and examined their behaviour in white matter. One line had lost white matter regulation of SOX10 induction, displaying homogeneous high SOX10 expression throughout the xenograft and was not analysed further. In contrast, the other four lines were similar to G144 cells, with a somewhat variable, but consistent increase in the proportion of SOX10^+^ cells in white matter as compared to grey matter (Fig. 2a, b and Supplementary Fig. 2d). In addition, as in G144 xenografts, SOX10^+^ cells proliferated significantly less than SOX10^−^ cells in white matter and partially progressed to CNP^+^ or CC1^+^ immature pre-oligodendrocyte cells (Fig. 2c and Supplementary Fig. 1k, l and Fig. 2e). To determine whether the white matter-induced response also occurs in primary patient tumours, we took two complementary approaches. First, we selected three SOX10^+^ cases based on retained SOX10 expression in derivative GSC lines (GCGR-L12, GL67 and GL23). We found that in all cases the white matter contained SOX2^+^ tumour cells, which upregulated SOX10 and proliferated less than their SOX10^-^ counterparts, confirming that GSC behaviour in xenograft models reflects disease phenotypes (Fig. 2d, e and Supplementary Table 3)^3^. SOX2 staining was specific to GBM cells, as the vast majority of endogenous SOX10^+^ OPCs were negative for SOX2 in areas of tumour infiltration (Supplementary Fig. 3a). Second, we selected 23 white matter-containing cases without prior knowledge of SOX10 status. Remarkably, we found that 15 cases contained at least a subset of SOX10^+^ cells in white matter, regardless of molecular characteristics (Fig. 2f, Supplementary 3b and Supplementary Table 3). In addition, SOX10^+^ cells were lowly proliferative, progressed to a CNP^+^/CC1^+^/MBP^−^ immature pre-oligodendrocyte state and were restricted to MBP^+^ tumour regions (Fig. 2e and Supplementary Fig. 3c–e). Finally, in a somatic *Nf1/Pten/p53* CRISPR-based mouse model, while the vast majority of tumour cells were SOX10^+^, a much greater proportion of tdTomato^+^ tumour cells differentiated to CC1^+^/CNP^+^/Ki67^−^ immature oligodendrocyte cells in the white matter relative to the grey matter (Supplementary Fig. 3f–j). These experiments confirm that a subset of GBMs progress to pre-oligodendrocyte-like cells in white-matter.

### Disrupted white matter drives pre-oligodendrocyte differentiation

**Fig. 3:**
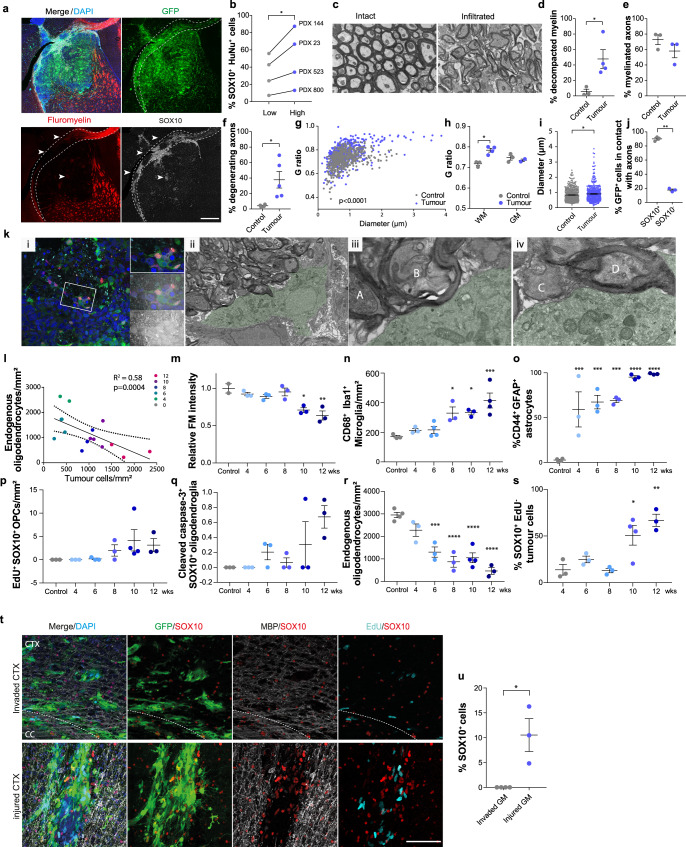
Exposure to disrupted myelin drives GBM progression down the oligodendrocyte lineage. **a** SOX10 (grey), fluoromyelin (FM, red), DAPI (blue) immunofluorescence of GFP^+^ G144 xenografts (PDX144). Arrowheads denote disrupted areas. Scale = 500 µm **b**, % SOX10^+^ tumour cells in areas of high (blue)/low (grey) disruption in indicated xenografts. ≥120 cells across ≥6 ROIs per region. *n* = 4 xenografts. p = 0.03, Mean, paired one-tailed Student’s t test **c**, Electron micrographs of myelin bundles in the ipsilateral (infiltrated) and contralateral (intact) striatum of PDX144. Scale=1 µm. **d**–**i**, quantification of indicated axonal and myelin phenotypes in EM data from **b**. *n* = 3-4 xenografts. **d** *p* = 0.03, f, *p* = 0.03, **g** *p* < 0.0001, **h** *p* = 0.01, **i** p = 0.01, **p < 0.01, *p < 0.05, Mean ± SEM, paired one-tailed Student’s t test, 2-way ANOVA or linear regression. **j** % GFP^+^ tumour cells in contact with axons. ≥300 SOX10^+^/SOX10^−^ cells across 3 ROIs. Mean^ ± ^SEM, *n* = 3 tumours, *p* < 0.0001. Unpaired two-tailed Student’s t test. **k** *i* Correlative light and electron micrograph (CLEM) of tumour cell directly interacting with multiple white matter axons. *ii-iv* magnifications of *i* highlighting interactions with decompacted myelin (A, B), naked (C), and intact myelinated axon (D) Scale = 10 µm (i), 5 µm (ii), 1 µm (iii, iv). **l**–**s** time-course analysis of tumour cell differentiation and glial response during corpus callosum (CC) infiltration in PDX144. **l** quantification of FM intensity relative to tumour density. Dots indicate individual xenografts colour-coded by time-point. *n* = 3 tumours/time point, *n* = 2 control brains. R^2^ = coefficient of determination. **m**–**s** quantification of indicated cell types over time. Minimum ROI = 300 µm. Mean ± SEM, *n* = 3 xenografts. **m** 10wk *p* = 0.005, 12wk *p* = 0.0009, **n** 8wk *p* = 0.02, 10wk *p* = 0.02, 12wk *p* = 0.0005, **o** 4wk *p* = 0.0009, 6wk *p* = 0.0003, 8wk *p* = 0.0002, 10wk *p* < 0.0001 12wk *p* < 0.0001, **r** 6wk *p* = 0.0003, 8wk *p* < 0.0001, 10wk *p* < 0.0001, 12wk *p* < 0.0001, **s** 10wk *p* = 0.01, 12wk *p* = 0.0014, one-way ANOVA with Dunnett’s multiple comparison tests. **t** SOX10 (red), MBP (grey), EdU (turquoise), DAPI (blue) immunofluorescence of GFP^+^ G144 cells directly injected (injured CTX) or invaded into the inner cortex from the tumour bulk (invaded CTX). Dotted lines delineate the corpus callosum (CC). Scale = 100 µm. **u** % SOX10^+^/EdU^-^ differentiated tumour cells shown in **t** ≥340 cells per xenograft. Mean ± SEM, *n* = 3 xenografts per group. *p* = 0.01. Unpaired two-tailed Student’s *t* test.

Next, we sought to understand what properties of white matter are differentiation-promoting. We noticed that SOX10 induction was most robust in areas where OSP staining appeared disrupted, suggesting that it might be linked to demyelination (Supplementary Fig. 1i). We therefore assessed myelin integrity in xenografts by fluoromyelin staining and by electron microscopy (EM). The proportion of SOX10^+^/HuNu^+^ tumour cells was highest in heavily infiltrated, fluoromyelin negative, white matter areas, as compared to dye-positive, low tumour-density, myelin regions in four independent xenografts (Fig. 3a, b and Supplementary Fig. 4a). A similar differentiation pattern was also found in patient tissue, where only few SOX2^+^ tumour cells upregulated SOX10 in intact myelin (Supplementary Fig. 4b). EM analysis of G144 xenografts further revealed severe axonal pathology and demyelination in highly infiltrated white matter regions, including axonal swelling and vacuolisation, presence of dark axons, myelin decompaction and a significant increase in g-ratios relative to contralateral normal brain (Fig. 3c–i and Supplementary Fig. 4c). In contrast, g-ratios remained normal in the tumour-infiltrated striatal grey matter (Fig. 3h), indicating that demyelination is specific to white matter. Consistent with this, activated microglia increased selectively in tumour-infiltrated white matter and endogenous oligodendrocytes were significantly decreased in the CC (Supplementary Fig. 4d–g). Furthermore, activated microglia with engulfed myelin debris was frequently observed in both xenografts (Supplementary Movie File 1) and human samples, and colocalised with SOX10^+^ tumour cells (Supplementary Fig. 4h–j). Surprisingly, despite severe myelin dysfunction, the mice did not exhibit overt motor or cognitive deficits.

Super resolution and correlative light and electron microscopy (CLEM) showed that within areas of myelin disruption tumour cells associated closely with both intact myelinated and demyelinating axons (Fig. 3j, k). This did not affect tumour cell survival (Supplementary Fig. 4k, l), but occasionally resulted in tumour cells acquiring oligodendrocyte morphology, in line with a differentiation response (Supplementary Fig. 4m).

Together, this data supports a model whereby tumour infiltration into the white matter induces an injury-like microenvironment, which in turn triggers GBM differentiation in a tumoursuppressive feedback loop. To test this more directly, we carried out a time-course analysis of the response of GBM cells and the CC microenvironment to tumour infiltration. G144 xenografts were collected at 2 weeks intervals from full engraftment at 4 weeks to symptomatic disease at 12 weeks post-implantation and subjected to immunohistochemistry for differentiation and activated glia markers. Tumour infiltration resulted in progressive loss of myelin integrity, which correlated with a gradual increase in astrocyte reactivity, microglia activation, OPC activation and oligodendrocyte death, all hallmarks of the glial response to brain injury (Fig. 3l–r, Supplementary Fig. 5a–d). Importantly, tumour cell differentiation paralleled these changes, with the number of SOX10^+^/EdU^−^ tumour cells gradually increasing over time and remaining lowly proliferative, indicative of stable pre-oligodendrocyte differentiation (Fig. 3s, Supplementary Fig. 5e).

To determine whether injury is causal to tumour differentiation, we next performed gain-of-function experiments. G144 cells were injected directly into the densely myelinated inner layers of the cortex, a brain region in which tumour cells frequently infiltrate from the striatal tumour bulk, but do not differentiate (Fig. 3t). Remarkably, the stab-wound injury caused by the needle (evidenced by induction of astrocyte reactivity and microglia activation, Supplementary Fig. 5f–h) was sufficient to induce GBM cell differentiation to SOX10^+^/EdU^−^ cells (Fig. 3t, u). Thus, GBM differentiation is a white matter injury-like response.

### Differentiation is niche-dependent

**Fig. 4:**
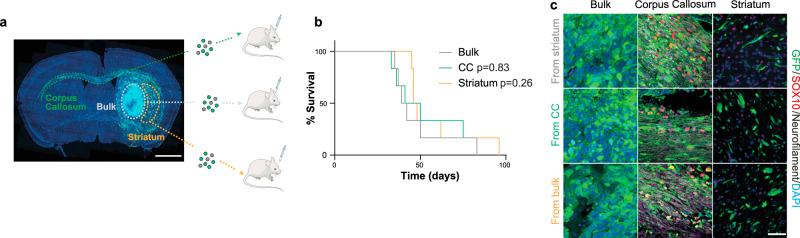
Differentiation depends on continuous exposure to white matter. **a** schematic representation of experimental workflow. **b** Kaplan Meier survival plot of nude mice injected with G144 cells acutely isolated from the bulk, corpus callosum (CC) and striatum of primary xenografts. *n* = 6 mice/group. **c** Representative images of secondary GFP^+^ tumours stained for neurofilament (grey) to identify axonal bundles, SOX10 (red) and DAPI (blue). Tumour areas from the indicated brain regions are shown revealing a differentiation pattern identical to primary lesions. Scale = 50 µm.

To determine the stability of white-matter induced differentiation, we carried out secondary xenografts. G144 cells were isolated from CC, ST and B xenograft regions as above, and immediately re-injected in the striatum of secondary hosts (Fig. 4a). Survival analysis indicated that all cells, regardless of region of origin, formed tumours with similar latency, indicative of comparable tumourigenic potential (Fig. 4b). Furthermore, independent of initial SOX10 levels, all secondary lesions recapitulated SOX10 expression patterns of the primary tumours, which was high in white matter and low in grey matter regions and bulk (Fig. 4c). This suggests that once removed from white matter, pre-oligodendrocyte tumour cells may de-differentiate back to a GSC state. To directly test this hypothesis, we used the surface marker O4 together with GFP to FACS-purify pre-oligodendrocyte tumour cells from myelin-rich CC and B regions of xenografts, seeded them acutely in vitro in the presence of mitogens and monitored their morphology and proliferation in real-time by time-lapse microscopy (Supplementary Fig. 6a, b). Consistent with the in vivo results, we found that approximately 20% of branched, pre-oligodendrocyte O4^+^ tumour cells retracted their processes, re-acquired GSC morphology, and re-entered the cell-cycle (Supplementary Fig. 6b and Supplementary Movie Files 2, 3). Thus, maintenance of pre-oligodendrocyte fate in vivo requires continuous exposure to white matter.

### SOX10 drives differentiation

**Fig. 5:**
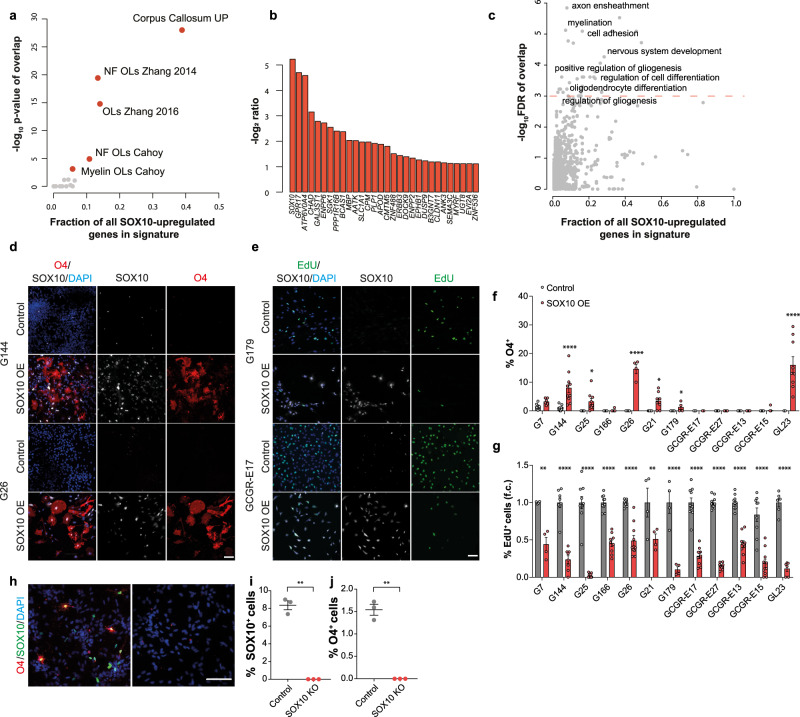
SOX10 overexpression induces pre-oligodendrocyte differentiation. **a** Enrichment of genes upregulated by SOX10 in vitro for signatures of glial and neuronal lineages (Supplementary data 4 and 5) and genes up-regulated in G144 xenografts upon invasion into the Corpus callosum (Corpus callosum UP). -log_10_ transformed p-values of one-sided Fisher tests plotted as a function of the percentage of all SOX10 regulated genes in each list. **b** most up-regulated genes upon SOX10 induction in vitro (top 20). DEseq2 log_2_ ratio of Doxycyclin- versus vehicle-treated cells are plotted. **c** GO enrichment analysis of the genes positively regulated by SOX10 in vitro (Supplementary data 4). GO terms belonging to the Biological process ontology are shown and selected terms are highlighted. -log10 transformed FDR q-values of overlaps are plotted as a function of the percentage of all SOX10 regulated genes present in each list. **d** O4 (red), SOX10 (grey) and DAPI (blue) staining of indicated GSC lines transduced with empty (Control) or constitutive SOX10-encoding lentiviral vectors. Scale = 100 µm. **e** representative images of indicated lines transduced with control or SOX10 lentivirus (SOX10 OE) and stained for EdU (green), SOX10 (grey) and DAPI (blue). Scale = 100 µm. **f** quantifications of % O4^+^ pre-oligodendrocyte cells in cultures shown in **d**. ≥200 cells across duplicate coverslips per biological repeat. Mean ± SEM, *n* = 3 independent transductions. G144 *p* < 0.0001, G25 *p* = 0.02, G26 *p* < 0.0001, G21 *p* = 0.04, GL23 *p* < 0.0001. Two-way ANOVA with Sidak’s multiple comparisons test. **g** quantifications of percentage of proliferating (EdU^+^) cells in the same cultures as in **e**. ≥200 cells across duplicate coverslips counted per biological repeat. Mean ± SEM, *n* = 3 independent transductions, G7 p < 0.0001, G144 *p* < 0.0001, G25 *p* < 0.0001, G166 *p* < 0.0001, G26 *p* < 0.0001, G21 *p* = 0.0004, G179 *p* < 0.0001, GCGR-E17 *p* < 0.0001, GCGR-E27 *p* < 0.0001, GCGR-E13 *p* < 0.0001, GCGR-E15 *p* < 0.0001, GL23 *p* < 0.0001. Two-way ANOVA with Sidak’s multiple comparisons test. **h** representative immunofluorescence images of Control and SOX10 knock-out (SOX10 KO) G144 cultures differentiated for 14 days by growth factor withdrawal and stained for SOX10 (green) and O4 (red). Scale = 100 µm. **i**, **j** quantifications of the cultures in **h**. ≥250 cells across duplicate coverslips per biological repeat. Mean±SEM, *n*  =  3 independent cultures, **i** *p* < 0.0001, **j** *p* = 0.0002. unpaired two-tailed Student’s t test.

In normal oligodendrogenesis, SOX10 is essential for lineage progression, differentiation and myelination^32–34^. We therefore asked whether the increase in SOX10 observed in white matter may also play a causal role in the induction of tumour cell differentiation. To address this, we transduced G144 cells with an inducible Tet-ON SOX10 overexpression construct and profiled vehicle- and doxycycline (Dox)-treated cultures by RNA-seq at 48 h post-induction. This time point was chosen to maximise the specificity of detected transcriptional changes and enrich for direct SOX10 targets. Immunofluorescence analysis confirmed detectable SOX10 protein expression in approximately 60% of the cells (Supplementary Fig. 6c, d). We identified 186 differentially expressed genes, the majority of which were upregulated (157, Supplementary data 4). Approximately 40% of SOX10-induced genes overlapped with genes upregulated in the corpus callosum in vivo, alongside significant overlap with signatures of oligodendrocyte lineage cells (Fig. 5a). These genes included both immature and mature oligodendrocyte markers, such as *GPR17*, *ERBB3* and the myelin genes *PLP1*, *CLDN11*, *MYRF* and *UGT8* (Fig. 5b). Furthermore, GO analysis identified enrichment for terms associated with oligodendrocyte differentiation, including axon ensheathment, myelination, cell adhesion and positive regulation of gliogenesis (Fig. 5c and Supplementary data 5). Thus, SOX10 controls a transcriptional programme of oligodendrogenesis in tumour cells.

To functionally assess effects of this programme on tumour cell phenotypes, we overexpressed SOX10 in a panel of patient-derived GSC lines. This included two lines with ability to spontaneously differentiate to pre-oligodendrocytes (G7, G144). Cells were transduced with lentiviral vectors expressing constitutive SOX10 (SOX10 OE), or control empty vectors, and subjected to differentiation by growth factor withdrawal for 7 days^35^. SOX10 overexpression induced 7 of the 12 lines to generate O4^+^ pre-oligodendrocytes, regardless of basal differentiation competency, and, remarkably, decreased proliferation in all lines, as measured by EdU incorporation (Fig. 5d–g). This indicates that SOX10 is sufficient for GBM cell differentiation. To determine if it is also necessary, we knocked-out *SOX10* by gene editing in G144 cells and carried out differentiation assays as above. We found that differentiation to O4^+^ cells was fully abolished in the absence of SOX10 (Fig. 5h–j). Together, these experiments demonstrate that SOX10 functions as a master regulator of pre-oligodendrocyte fate in GBM and confirm that corpus callosum phenotypes in vivo are largely mediated by SOX10 upregulation.

### Increased SOX10 suppresses tumourigenesis

**Fig. 6:**
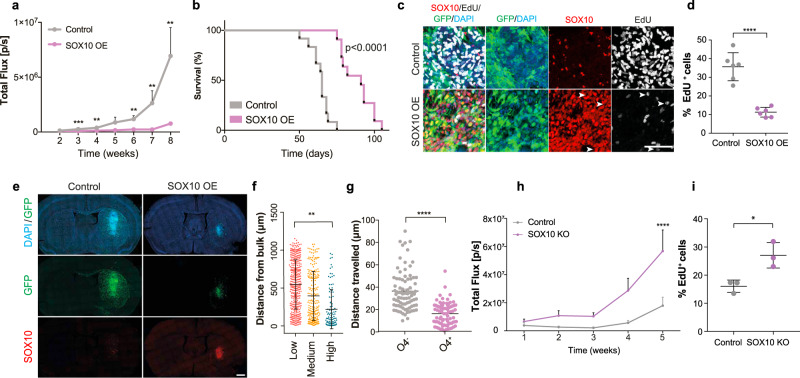
SOX10 overexpression delays tumourigenesis. **a** quantification of luciferase bioluminescence radiance measured at the indicated time points in Control G144 and SOX10 overexpressing (SOX10 OE) xenografts. *n* = 12 for Control and 13 for SOX10 OE, error bars denote SEM. Unpaired two-tailed Student’s t test. **b** Kaplan Meier survival plot of mice injected with GFP^+^ G144 Control and SOX10 OE cells. *n* = 8 for Control and 10 for SOX10 OE, *p* < 0.0001. **c** SOX10 (red), EdU (grey) and DAPI (blue) immunofluorescence staining of GFP^+^ Control and SOX10 OE tumours at 4 weeks post-injection. Arrowheads denote EdU^+^ cells that are negative for SOX10. Scale = 100 µm. **d** % EdU^+^/GFP^+^ tumour cells in Control and SOX10 OE tumours from c. ≥200 cells were quantified across 2 ROIs/mouse. Mean ± SEM are plotted. *n* = 3 mice/group *p* = 0.0006. Unpaired two-tailed Student’s t test **e**, representative images of GFP^+^ G144 Control and SOX10 OE tumours stained for SOX10 (red) and DAPI (blue) at 4 weeks post-injection. Scale = 1 mm. **f** quantification of migrated distance from tumour bulk edge of tumour cells expressing low, medium or high SOX10 levels in SOX10 OE tumours at 4 weeks post-injection. ≥200 cells were quantified per mouse. Each dot represents a cell. Mean ± SEM are indicated. *n* = 3 mice/group. One-way ANOVA. **g** migrated distance of O4^+^ and O4^−^ tumour cells acutely isolated from the corpus callosum region of G144 xenografts (PDX144) and cultured for 72 h in neural stem cell conditions. Each dot represents a cell, *n* = 90 cells/group pooled from 3 independent experiments. Median±SEM are shown. *n* = 3 tumours *p < 0.05. Unpaired two-tailed Student’s t test. **h** quantification of luciferase bioluminescence radiance of Control and SOX10 knock-out (SOX10 KO) PDX144 measured at the indicated time points. *n* = 5 Control and *n* = 7 SOX10 KO tumours. Mean ± SEM are indicated. Unpaired two-tailed Student’s t test. **I** % EdU+ cells in Control and SOX10 KO PDX144 at 4 weeks post-implantation.

Our results so far suggest that manipulations capable of increasing SOX10 levels niche-independently should suppress tumourigenesis by promoting stable differentiation. To test this more directly, we performed intracranial transplantations of luciferase and GFP-tagged G144 cells transduced with SOX10 OE or Control lentiviruses and monitored tumour growth and disease-free survival. SOX10 overexpression significantly delayed tumourigenesis, resulting in an overall increase in median survival from 65 to 91 days (Fig. 6a, b). To understand the mechanisms responsible, we examined Control and SOX10 OE tumours at 4 weeks post implantation by immunofluorescence analysis. EdU labelling revealed a marked decrease in the total number of proliferating tumour cells in SOX10 OE compared to Control lesions (Fig. 6c, d). Importantly, time-course analysis of SOX10 levels in the tumours that eventually formed revealed that SOX10 overexpressing cells were progressively outcompeted by cells that had escaped transduction, confirming that high SOX10 levels are tumoursuppressive (Supplementary Fig. 6e).

Although SOX10-mediated differentiation was most pronounced in invasive tumour cells that infiltrate the corpus callosum (Fig. 1e), normal oligodendrocyte maturation is accompanied by a progressive loss of migratory potential^36,37^. We therefore sought to understand the impact of differentiation on GBM invasion by imaging whole sections of 4 weeks tumours. Surprisingly, SOX10 OE tumours appeared less diffuse and migrated much shorter distances than controls (Fig. 6e and Supplementary Fig. 6f). In addition, within SOX10 OE tumours the majority of invasive tumour cells had low or undetactable SOX10 levels, whereas SOX10 high cells were found predominantly closest to the tumour bulk (Fig. 6f). These results indicate that differentiation also reduces the migration of GBM cells. To determine whether this phenotype was cell-intrinsic, we measured in vitro motility of SOX10 OE and Control G144 cells by live cell imaging and found that increased SOX10 levels profoundly reduced cell motility (Supplementary Fig. 6g–i). This was not an artefact of SOX10 overexpression, as O4^+^ tumour cells acutely FACS-purified from the corpus callosum of wildtype G144 xenografts were also significantly less motile than O4^-^ cells from the same region (Fig. 6g).

These results suggest that white matter effects are tumour suppressive and might slow down the progression of the primary disease. To test this hypothesis experimentally, we compared the tumourigenicity of parental and SOX10 knock-out G144 cells, in which differentiation along the oligodendrocyte lineage is abolished (Fig. 5h–j). SOX10 knock-out tumours had a significantly faster growth rate than controls, as measured by longitudinal bioluminescence imaging and analysis of EdU incorporation (Fig. 6h–i). These experiments demonstrate that white matter-driven pre-oligodendrocyte maturation suppresses tumourigenesis by inhibiting proliferation and invasion of GBM cells and that in the absence of this response GBMs are more aggressive.

**Fig. 7:**
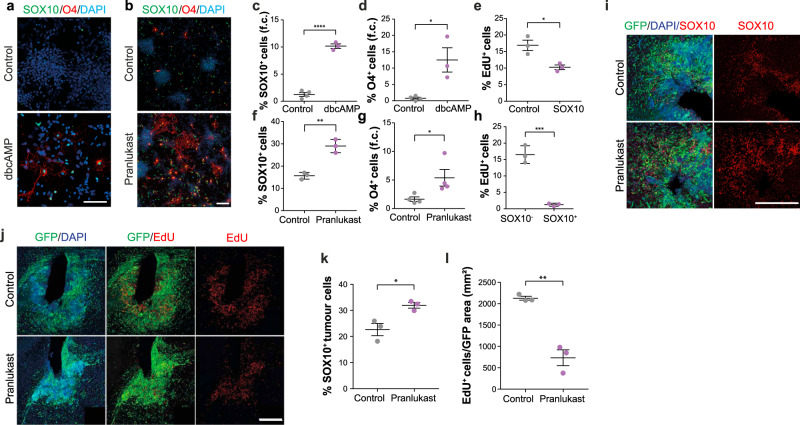
Myelination-promoting compounds suppress tumour growth. **a** representative images of untreated and dibutyril cAMP-treated (dbcAMP) G144 cultures stained for SOX10 (green), EdU (grey), O4 (red) and DAPI (blue). Scale = 100 µm. **b** O4 (red), SOX10 (green) and DAPI (blue) staining of G144 cultures treated with vehicle (Control) or Pranlukast. Scale = 50 µm. **c**–**e** quantification of cultures in a, showing percentages of indicated populations before and after treatment. Fold change relative to control cultures (f.c.) is shown in **c** and **d**. ≥1000 cells across duplicate coverslips were counted per biological repeat. Mean ± SEM, *n* = 3 independent cultures, **a** *p* < 0.0001, **b** *p* = 0.01, **c** *p* = 0.02. Unpaired two-tailed Student’s t test. **f**–**h** quantification of cultures in b, showing percentages of indicated populations before and after treatment. Fold change relative to control cultures (f.c.) is shown in g. ≥800 cells across duplicate coverslips were counted per biological repeat. Mean ± SEM, *n* = 3 independent cultures. **c** *p* = 0.002, **d** *p* = 0.05, **e** 0.0006. Unpaired two-tailed Student’s t-test. **i**, **j** representative immunofluoresce images of DMSO- (Control) and Pranlukast-treated GFP^+^ G144 xenografts (PDX144) stained for SOX10 (red) and EdU (red). Scale = 500 µm. **k**, quantifications of number of SOX10^+^ and **l**, EdU^+^ tumour cells in the xenografts shown in **i** and **j**. Scale = 500 µm. Mean ± SEM, n = 3 xenografts. **t** *p* = 0.02, **u** *p* = 0.002. Unpaired two-tailed Student’s t test. *n* = 3 xenografts per group. Unpaired two-tailed Student’s t test.

Finally, we explored the translational potential of these findings by testing whether known myelination-inducing pharmacological agents could differentiate GBM cells by increasing endogenous SOX10 levels. We first treated cultured G144 cells with two compounds: the clinically-approved anti-asthma medication Pranlukast and the cell permeable cAMP analogue dibutyril cAMP (db-cAMP). In addition to blocking cysteinyl-leukotriene 1 receptor, Pranlukast inhibits GPR17, a negative regulator of oligodendrocyte development^38–40^. GPR17 is normally expressed in immature oligodendrocytes^38^ and, importantly, was strongly induced in G144 cells purified from the CC and upon SOX10 overexpression (Figs. 1d and 5b). db-cAMP treatment of normal progenitors results in elevated intracellular cAMP levels, which promote oligodendrocyte differentiation, likely by phenocopying GPCR activity^41,42^. Treatment of G144 with either compound for 2 weeks was sufficient to increase endogenous SOX10 levels and differentiation to O4^+^ pre-oligodendrocyte cells, leading to a reduction in proliferation (Fig. 7a–h). These effects were fully dependent on SOX10, as no differentiation was observed in drug-treated SOX10 knock-out cells (Supplementary Fig. 7a, b). Next, we administered Pranlukast to tumour-bearing mice in vivo. As Pranlukast has poor BBB penetration^43^, we delivered it intrathecally using osmotic mini-pumps. Strikingly, we found a significant increase in the number of SOX10^+^ cells in Pranlukast-treated tumours, which was accompanied by a dramatic reduction in proliferation relative to saline-treated controls (Fig. 7i–l). Pranlukast did not affect endogenous inflammatory glia, indicative of a direct effect on the tumour cells (Supplementary Fig. 7c–e). We conclude that a subset of GBMs can be induced to undergo pre-oligodendrocyte differentiation with small molecules that raise SOX10 levels and propose that such differentiation therapy may be an effective strategy for curtailing tumourigenesis and recurrence.

## Discussion

The failure of both conventional and targeted therapies is commonly attributed to the pervasive molecular intra- and intertumoural heterogeneity of GBM, which occurs at the genetic, epigenetic, transcriptional and functional levels^44–51^. Despite this staggering diversity however, recent studies are beginning to reveal that all GBMs converge on a finite number of cellular states, which recapitulate normal developmental programmes^8,9^. Such convergence offers hope of identifying shared biological vulnerabilities that could be exploited for the treatment of many patients, independent of genetic diversity. Here we identified one such vulnerability: the competence of GBM cells to undergo pre-oligodendrocyte differentiation.

Although GSCs retain ability to partially differentiate into mature glial cells in vitro, the extent to which this occurs in tumours has remained unclear^8–10,52^. Our results demonstrate the existence of robust differentiation in vivo, including in patient material, which is controlled by the microenvironment. They further reveal that differentiation occurs in specialised niches within the brain and identify the white matter as one such pro-differentiative niche for tumour cells with oligodendrocyte competency^30^. Thus, despite the presence of extensive genetic abnormalities, exposure to appropriate environmental cues is sufficient to revert GBM cells to a more normal, differentiated phenotype, underscoring the dominance of the microenvironment in suppressing malignancy^53,54^.

We found that differentiation is an injury-like response, which results from infiltrating tumour cells disrupting the white matter. Thus, although invasion along white matter is a common mode of infiltration, the ensuing myelin damage creates a tumour-suppressive feedback loop, which paradoxically slows GBM spread. Interestingly, the microenvironment of tumour-infiltrated white matter appeared remarkably similar to that of neuroinflammatory disease states, entailing severe demyelination and axonal pathology^55^. Our EM analysis suggests that in the tumour context demyelination may be caused by both oligodendrocyte and neuron death. The underlying mechanisms remain to be determined, but a combination of compression injury to the axons, microglia and astrocytes activation, as well as excitotoxins are all likely to contribute^55,56^.

The finding of OPC activation in tumour-infiltrated white matter suggests that the ensuing demyelinating microenvironment initiates a repair programme in normal glia. It is therefore tempting to speculate that GBM cells may recapitulate a similar oligodendrogenic programme, which results in their partial differentiation to pre-oligodendrocyte cells. While it is clear that an injury-like microenvironment drives differentiation, the signals and cell types responsible remain undefined. These are likely to be complex and combinatorial, but our results suggest that microglia, a key regulator of normal re-myelination, may play an important role^57^. Indeed, we found that microglia activation was selective to tumour white matter and immediately preceded tumour cell differentiation. It would be of great interest to examine the contribution of microglia to GBM differentiation in future studies.

Our findings are of clinical relevance as we show that exploiting the aberrant GBM injury-like response through niche-independent upregulation of SOX10, locked tumour cells in the differentiated, non-proliferative state and suppressed tumourigenesis in preclinical models of the disease. Furthermore, our work predicts that myelination-promoting compounds, should be particularly effective in tumours with oligodendrocyte lineage competency, providing a potential strategy for patient stratification. Future studies should continue to explore how the intersection of repair and developmental programmes may afford novel translational opportunities for GBM.

## Methods

### Animals

All procedures were performed in compliance with the Animal Scientific Procedures Act, 1986 and approved by the UCL Animal Welfare and Ethical Review Body (AWERB) in accordance with the International guidelines of the Home Office (UK). C57Bl6 and immunocompromised mouse lines were purchased from Charles River.

### Patient-derived xenograft models

Xenografts were performed using CD-1 nude mice for G144 and NOD-SCID-IL2R gamma chain-deficient (NSG) for all other lines. For all tumour studies 8–12 week old female immunocompromised mice underwent stereotactic implantation of GSC lines using 1 × 10^5^ cells with the exception of G144 reinjection experiments for which 5 × 10^4^ were used (anteroposterior 0, mediolateral -2.5, dorsoventral -3.5; corpus callosum: anteroposterior 0, mediolateral -1, dorsoventral -1.75; inner cortex: anteroposterior 0, mediolateral -1, dorsoventral -1; outer cortex: anteroposterior 0, mediolateral -1, dorsoventral -0.5). Tumour growth was monitored using an IVIS Spectrum in vivo imaging system (Perkin Elmer). A total of 10 min following i.p. D-luciferin (120 mg/kg, Intrace medical) bioluminescent images were acquired under isoflurane anaesthesia. Tumour size was quantified by calculating total flux (photons/s/cm^2^) using Living Image software (Xenogen, Caliper Life Sciences). Animals were sacrificed and tumours collected when they showed signs of distress or >10% weight loss. For experiments shown in Fig. 4c–f and r–u tumours were collected at 4 weeks post-implantation. To assess tumour cell proliferation EdU was administered by i.p. injection (50 mg/kg) 4 h prior to collection. Survival was analysed using the Kaplan–Meier method and significance calculated using the log-rank Mantel–Cox test. For all models GBM tumour samples were obtained from consenting patients, following local ethical board approval from the Research Ethics Board at The Hospital for Sick Children (Toronto, Canada), or from London - Queen Square Research Ethics Committee (08-077).

### De novo models

De novo models were generated using a CRISPR/Cas9-based deletion of *Nf1*, *Pten* and *Trp53* tumour suppressors^58^. tdTomato encoding piggybac transposons were co-delivered to fluorescently label resulting tumours. Briefly, P2 C57Bl/6 pups underwent intraventricular plasmid administration using an Eppendorf Femtojet Microinjector. Electroporation was performed using tweezertodes positioned on either side of the pup’s head delivering 5 square pulses (100 V, 50 ms pulse ON, 850 msec pulse OFF) delivered by the Gemini BTX electroporator.

### Cell culture

Patient-derived GCGR cell lines were provided by the glioma cellular genetics resource: (www.gcgr.org.uk). All GSC lines were cultured adherently in serum-free GSC media (N2 (1/200), B27 (1/100) (Life Technologies), 1 mg/ml laminin (Sigma), 10 ng/mL EGF and FGF-2 (Peprotech), 1× MEM NEAA (Gibco), 0.1 mM betamercaptoethanol, 0.012% BSA (Gibco), 0.2 g/L glucose (Sigma), 1000 U/ml penicillin-streptomycin (Sigma))^25^. Medium was changed every 3 d, and cells were dissociated using Accutase solution (Sigma). For induction of SOX10 expression, doxycycline was used at a concentration of 3 μg/ml. For experiments involving assessment of proliferation, cells were exposed to 10 μm EdU for 4 h prior to collection.

### FACS of tumour cells

Brains were harvested and tumour regions corresponding to tumour bulk (B), invaded striatum (ST) and corpus callosum (CC) were micro-dissected under fluorescent guidance in ice-cold HEPES buffered HBSS. Micro-dissected regions were dissociated by incubating with papain (20 units/ml) DNase (0.005%) for 30 min at 37 °C (Worthington). Samples were triturated in HEPES buffered EBSS to fully dissociate the tissue and centrifuged (3 min, 300 × *g*). Digestion was terminated by trituration in EBSS ovomucoid inhibitor (10 mg/ml), albumin (10 mg/ml) and DNase (0.005%). Following centrifugation (3 min, 300 × g), cells were resuspended in 400 μL FACS buffer (1.5% BSA, 2.5 mM HEPES, 1 mM EDTA in PBS) containing 2.5% RNAsin and 1/10000 DAPI (Promega). Cells for re-injection experiments were isolated as above and subjected to a further two centrifugation steps (1 min at 200 × *g*) in 10 ml warm EBSS after digestion to reduce myelin debris. For isolation of O4^+^ tumour cells, cells were dissociated as described above. Following centrifugation in EBBS, cells were resuspended in 500 μl GSC media containing 1:500 mouse anti-O4 (Alexa Fluor 594) and incubated for 15 min at 37 °C. Cells were washed once in PBS + 3% BSA and resuspended in 400 μL FACS buffer containing 1/10000 DAPI. O4^+^ and O4^-^ populations were seeded in serum-free GSC media containing EGF and FGF for live-cell imaging.

### Whole-transcriptome amplification, library construction, sequencing and processing

For RNA-seq of FACS-sorted tumour cells, dsDNA libraries were prepared according to the Smart-seq2 protocol from FACS-sorted GFP + cells (RIN > 8)^59^. Next-generation sequencing libraries were prepared from 1 ng dsDNA using the Nextera XT DNA library preparation kit (Illumina) and indexed using the Nextera XT index Kit (Illumina).

For RNA-seq of in vitro GSCs, oligo dT-based mRNA isolation was performed using the NEBNext Poly(A) mRNA magnetic isolation module. dsDNA libraries were prepared using NEB Ultra II directional RNA library prep kit and indexed using NEBNext^®^ Multiplex Oligos. All libraries were diluted to a final concentration of 2.5 nM, pooled and sequenced on an Illumina HiSeq 2500 instrument. Raw data were processed using RTA version 1.18.64, with default filter and quality settings. The reads were demultiplexed with CASAVA 1.8.4 (allowing 0 mismatches). Raw reads were aligned to the human genomes (hg38) and assigned to genomic features both using the STAR aligner^60^. After filtering, only genes with at least 5 counts in at least 3 samples were included in the final dataset (*n* = 16044 for in vivo RNA-seq, *n* = 14621 for in vitro RNA-seq).

### Analysis of RNA-seq data

Differential expression analysis was performed and normalized counts were generated using the DESeq2 Bioconductor package (Supplementary Table 1)^61^. For Fig. 1c, genes significantly regulated (P_adjust_ < 0.1) and with an absolute DESeq2 log2 ratio >0.58 in at least one of the Corpus callosum vs Bulk or Striatum vs Bulk comparisons were selected and clustered using K-means (7 clusters). Overlap of each cluster with gene signatures specific for proliferating cells and a series of brain cell types was assessed using a one-sided Fisher exact test and p-values were corrected for multiple testing using the Benjamini-Hochberg approach. For Supplementary Fig. 1d, genes belonging to each gene signatures and significantly regulated (*p*_adjust_ < 0.1) in each comparison are shown. Brain signatures were generated using one human and two mouse transcriptomics datasets^26–28^. Genes with multiple entries were averaged and low coverage genes filtered out. Condition replicates were averaged, data median centred before the most variable genes between cell types were selected (Coefficient of variation, CV > 1 for Zhang 2016, CV > 1.5 for Zhang 2014, CV > 0.1 for Cahoy 2008) for K-means clustering (Supplementary Fig. 1a–c). The cut-offs on coefficient of variation and the numbers of K-means clusters were selected empirically to maximise the discrimination between cell types. We defined single clusters with good cell type discrimination as gene signatures (Supplementary Fig. 1a–c, Supplementary data 2). Proliferating cells signatures were obtained from the GSEA website. Gene expression z-scores of the SOX10 mRNA were downloaded from cBioportal for samples from the TGCA cohort. GBM subtypes for each sample were retrieved from Wang et al.^50^. For Fig. 3c, GO enrichment analysis was performed using the VLAD on-line portal (http://proto.informatics.jax.org/prototypes/vlad/).

### SOX10 overexpression

Constitutive SOX10 expression for analysis of differentiation and proliferation in vitro as well as in vivo tumorigenesis studies was achieved by gateway cloning of human SOX10 from pDONR221-SOX10 into a pLenti-CMV-BLAST-DEST vector. For the empty control vector, the ccdB gene was removed using EcoRV and HpaI blunt-end ligation. For in vitro RNA-seq an inducible SOX10 construct was generated using gateway cloning to insert SOX10 from pDONR221-SOX10 into pCW57.1. pDONR221-hSOX10 was a gift from William Pavan (Addgene plasmid #24749; http://n2t.net/addgene:24749; RRID:Addgene_24749)^62^. pCW57.1 was a gift from David Root (Addgene plasmid #41393; http://n2t.net/addgene:41393; RRID:Addgene_41393). Lentivirus was produced by cotransfecting with the HIV-1 packaging vector Delta8.9 and the VSVG envelope glycoprotein into 293 T cells using *polyethylenimine*. Virus was concentrated by ultracentrifugation (3 h, 50,000 × *g*, 4 °C).

### Cell migration assay

Live cell imaging was performed using either the Zeiss Live Cell Imager Z1 or the IncuCyte Zoom. Cells were tracked manually in ImageJ using the ManualTracking plugin, migration distance was calculated and cell trajectories were visualized using the Chemotaxis and Migration Tool provided by Ibidi.

### Quantitative RT-PCR

RNA was extracted using Trizol Reagent (Sigma). RNA was reverse transcribed using iScript gDNA clear cDNA synthesis kit (Bio-rad) and quantitative PCR was performed using the qPCRBIO SyGreen Mix Lo-Rox (PCR Biosystems). Relative expression values for each gene of interest were obtained by normalizing to GAPDH. Primers used are detailed in Supplementary Table 4.

### Immunofluorescence and Immunohistochemistry

For immunohistochemical analysis, mice were perfused with 4% PFA and the brain post-fixed in 4% paraformaldehyde overnight at 4 °C. 50 μm vibratome sections were permeabilised and blocked in 1% Triton X-100, 10% serum for 1.5 h then incubated overnight at 4 °C with primary antibodies diluted in 0.1% Triton X-100, 10% serum. For paraffin-embedded sections, following deparaffinisation, antigen retrieval was performed using citrate buffer (Sigma) and sections were permeabilised and blocked 1% Triton X-100, 10% serum for 1 hr then incubated overnight at 4 °C with primary antibodies diluted in 0.1% Triton X-100, 10% serum.

For in vitro immunofluorescence, cells were fixed in 4% PFA, permeabilised in 0.5% Triton X-100 and blocked in 10% serum in PBS then incubated with primary antibody overnight at 4 °C in PBS + 10% serum. Anti-O4 antibody staining was performed on live cells for 30 min at 37 °C in cell culture media. Secondary antibodies were diluted in 10% serum in DAPI (1:10000 in PBS) and incubated at RT for 1 h. Analysis of EdU incorporation was performed using Invitrogen’s^TM^ Click/iT^TM^ EdU imaging Kit according to the manufacturer’s specifications. Imaging was carried out using the Zeiss Z1 upright microscope or the Zeiss LSM880 confocal microscope. Quantification was performed using Fiji ImageJ.

Primary antibodies used were mouse anti-CC1 (1:1000, Abcam ab16794), rabbit anti-Ki67 (1:250, Abcam ab16667), goat anti-MBP (1:1000, Santa Cruz sc-13912), rat anti-MBP (1:500 paraffin; 1:1000 coverslips, Sigma MAB386), chicken anti-neurofilament (1:2000, Abcam ab4680), mouse anti-HuNu (1:250, Sigma MAB1281), mouse anti-O4 (1:500, R&D MAB1326), rabbit anti-RFP (1:500, Antibodies Online AA234 (ABIN129578)), rabbit anti-OSP (1:500, Abcam ab53041), goat anti-SOX10 (1:1000, R&D AF2864), rat anti-CD68 (1:500, Abcam ab53444), rabbit anti-Iba1 (1:1000, Wako 019-19741), mouse anti-Sox2 (1:100, Abcam ab79351), rabbit anti-Sox2 (1:1000, Abcam ab97959), rat anti-CD44 (IM7) (1:500, Invitrogen 14-0441-81), rabbit anti-GFAP (1:1000, Dako Z0334), mouse anti-CNP (1:500 Abcam ab6319). Alexa Fluor conjugated secondary antibodies were obtained from Thermo Fisher.

### Image processing and quantifications

All image quantifications were carried out using Fiji ImageJ. For analysis of SOX10 expression in infiltrating G144 tumour cells, white matter (corpus callosum) and grey matter regions containing at least 750 HuNu^+^cells were defined for quantification across *n* = 3 mice and SOX10^+^ and SOX10^-^ human cells counted within these regions. For analysis of EdU incorporation ≥500 HuNu^+^ cells were quantified for SOX10 expression and EdU positivity across 2 independent ROIs of infiltrated corpus callosum. For analysis of SOX10^+^/EdU^−^ cells upon implantation into the white matter of the corpus callosum (*n* = 3) or grey matter of the upper cortex (*n* = 3) a minimum of 140 cells per xenograft were counted. For analysis of regional EdU^-^ incorporation in GFP^+^ tumour cells of G144 xenografts (*n* = 3), ≥200 cells were counted per xenograft. For analysis of the impact of PDGFRA signalling on oligodendroglial differentiation in vitro, ≥3500 cells were counted per biological repeat (*n* = 4). For quantification of the percentage of SOX10^+^/HuNu^+^ tumour cells in white and grey matter of all other patient-derived xenografts *n* = 4, ≥550 HuNu^+^ cells were quantified across 2 independent ROIs within white matter (corpus callosum) or grey matter. Analysis of tumour cell proliferation within the corpus callosum of xenografts PDX 23, 67, 523 and 800 was carried out for ≥500 SOX10^+^ or SOX10^−^ HuNu^+^ cells across 2 independent ROIs. For analysis of SOX2^+^ tumour cells undergoing proliferation (Ki67^+^) in patient tissue, ≥300 cells were quantified across 2 independent ROIs for each case *n* = 3.

Quantification of the percentage of differentiated CC1^+^ tumour cells in white and grey matter of *Nf1/Pten/p53* mouse tumours was performed across 2 independent ROIs selected within white or grey matter and counting ≥700 cells per ROI. For analysis of percentage of proliferating (Ki67^+^) CC1^+^ and CC1^−^ tumour cells within the corpus callosum, ≥400 cells were quantified across 2 independent ROIs for *n* = 3 mice.

For quantification of percentage of SOX10^+^ tumour cells in areas of high and low myelin disruption, ≥120 cells were quantified across ≥6 ROIs selected within intact or disrupted white matter for *n* = 4 xenografts. Myelin disruption was defined as white matter regions containing Neurofilament^+^ axons and Fluromyelin staining intensity <25% of contralateral intact regions. For analysis of endogenous oligodendrocytes (SOX10^+^/HuNu^−^) within the corpus callosum as a function of number of invaded SOX10^+^ G144 (SOX10^+^/HuNu^+^) tumour cells, quantification was carried out across ≥2 ROIs and counting >1000 cells per mouse for n = 5 tumours and *n* = 2 intact control brains.

For quantification of the percentage of GFP^+^ tumour cells in contact with axons within these regions a minimum 300 SOX10^+^ or SOX10^−^ cells were quantified across 3 independent ROIs for *n* = 3 tumours. Contact was defined as <1.5 µm from the edge of the nucleus to a Neurofilament^+^ axon. For analysis of microglia within the white matter (corpus callosum), grey matter (cortex) or tumour bulk ≥140 cells per region per xenograft were counted. Analysis of endogenous oligodendrocytes was conducted across 5 xenografts and 2 control brains. ≥450 cells were counted across 2 ROIs/xenograft. Quantifications of cleaved caspase-3 staining of GFP^+^ G144 tumour cells within the white matter (corpus callosum), grey matter (cortex) or tumour bulk were conducted for ≥200 cells per region per xenograft (*n* = 3).

For timecourse analysis of corpus callosum fluromyelin intensity, mean grey values were normalised to the max grey values to account for variation in image intensity. For all timecourse analyses a minimum area of 300 µm^2^ was analysed for ≥3 xenografts.

For analysis of SOX10^+^ EdU^−^ cells within the invaded of injured cortex ≥340 cells were counted per xenograft (*n* = 3–4). For analysis of microglia within the invaded or injured cortex ≥90 cells were counted.

For quantification of the percentage of GFP^+^ tumour cells in contact with axons within these regions a minimum 300 SOX10^+^ or SOX10^−^ cells were quantified across 3 independent ROIs for *n* = 3 tumours. Contact was defined as <1.5 µm from the edge of the nucleus to a Neurofilament^+^ axon.

For quantifications of the percentage of O4^+^ pre-oligodendrocyte cells and EdU^+^ proliferating cells in control and SOX10 transduced cultures of GSC lines ≥200 cells across duplicate coverslips were counted per biological repeat for *n* = 6 independent cultures per line.

For analysis of differentiation in SOX10 knock-out cells ≥250 cells were counted across duplicate coverslips per repeat. For SOX10 induction in vitro ≥90 cells per group across 2 independent cultures on triplicate coverslips were counted.

For quantifications of the relative proportions of SOX10^−^, low or high tumour cells at different timepoints (pre-implantation, 4 weeks and survival (11–12 weeks) ≥270 cells were analysed. SOX10 status was assessed using mean grey values of individual nuclei. SOX10- cells were counted as those with a mean grey value less than the background. SOX10 high threshold was determined by measuring the minimum mean grey value of >20 cells showing high SOX10 expression.

Proliferation of control or SOX10 OE tumours was quantified across *n* = 3 tumours at 4 weeks post-implantation, counting ≥200 cells from 2 ROIs per mouse. Quantification of invasion of *n* = 3 tumours was performed by measuring the total area occupied by tumour cells and normalising it to the perimeter of the tumour bulk to account for different rates of tumour growth. Invasion was further analysed within SOX10 OE by measuring the distance of SOX10 low, medium and high expressing cells from the tumour bulk. SOX10 low and high threshold intensities were defined as the 25th and 75th percentiles respectively based on analysis of the average intensities across >100 nuclei within the tumour bulk ≥200 cells were quantified per mouse.

For in vitro quantifications of the percentage of O4^+^ and EdU^+^ cells following exposure to pranlukast or control, ≥800 cells across duplicate coverslips were counted for *n* = 3 independent cultures. For dbcAMP experiments, ≥900 cells were counted across duplicate coverslips for *n* = 3 independent cultures.

For in vivo quantifications of the percentage of SOX10^+^ GFP^+^ tumour cells in control or pranlukast treated tumours ≥1200 cells were counted. For analysis of proliferation total EdU^+^ nuclei were normalised to the total GFP + tumour area (*n* = 3). For analysis of microglia ≥420 cells were counted across *n* = 3 xenografts.

### Targeted electron microscopy

GFP-labelled G144 cells were injected into the striatum of immune-compromised mice and once tumours had developed, brains were perfusion fixed with 4% formaldehyde, and then further immersion fixed for 8 h. Vibrating microtome sections (100 µm) were immunolabelled for Neurofilament and SOX10 and imaged by confocal to map the location of the tumour within the section. For correlative light and electron microscopy (CLEM) of specific SOX10 positive GFP labelled cells, vibratome sections were mapped using the ×20 objective to identify regions of interest. A small asymmetric piece of tissue (<1 mm) containing the region of interest was further dissected from the vibratome section and was completely mapped using the ×63 objective. Vibratome sections were then processed for electron microscopy essentially as follows^63^. Sections were further treated sequentially with formaldehyde:glutaraldehyde, osmium tetroxide: potassium ferricyanide, osmium tetroxide, thiocarbohydrazide, uranyl acetate and lead aspartate prior to dehydration through an ethanol series and embedding in Epoxy resin. For CLEM samples, care was taken to ensure that the piece of tissue was mounted flat, in the correct orientation, to guarantee targeting the cells of interest imaged by confocal microscopy. Serial ultrathin sections (70 nm) were taken using a diamond knife (Diatome) and an ultramicrotome (UC7, Leica) and collected on formvar coated slot grids. Sections were imaged in a transmission electron microscope (T12 BioTwin, Thermofisher) and captured with a CCD camera running iTEM software (Morada, Olympus SIS). All EM analysis was conducted on ≥50 axons (*n* = 3–4). Axons were considered to have decompacted myelin when >15% of the axonal circumference exhibited decompaction of the associated myelin. Degenerating axons were scored as those exhibiting any of the following features: condensed axoplasm, organelle accumulation, axonal swelling, vacuoles, dark axoplasm. G ratios were calculated by dividing the axonal diameter by the corresponding axonal + myelin sheath diameter. Feret diameters were used to account for the imperfect circularity of axons.

### Neuropathological assessment of SOX10 expression in human GBM

SOX10 expression pattern was investigated in brain tissue samples of 26 glioblastoma patients operated on at the National Hospital for Neurology and Neurosurgery, UCL Hospitals Foundation Trust between 2009 and 2017. None of the patients had radiotherapy or chemotherapy prior to surgery, and they did not have any relevant comorbidities. Patients consent was obtained for the use of all samples. The project received ethical approval from London—Queen Square Research Ethics Committee (08-077). Clinical information is provided in Supplementary Table 3.

For each case, the resection material was reviewed and the region containing tumour infiltration in the surrounding white matter was selected for the analysis. The resected tissues were immediately fixed in 10% buffered formalin and processed into paraffin blocks using standard methods in the Division of Neuropathology, NHNN.

### Statistics

Statistical analysis was performed using GraphPad Prism 7.0. All data are expressed as mean ± SEM. Significance was calculated using 1- or 2-tailed Student’s *t* test, ANOVA with Bonferroni post-hoc test, Two-way ANOVA with Sidak’s multiple comparisons tests or Pearson’s correlation as indicated in the figure legends. No statistical method was used to predetermine sample size. Sample size was determined based on existing literature and our previous experience. Shapiro–Wilk test was used to confirm the normal distribution of the data.

### Reporting summary

Further information on research design is available in the Nature Research Reporting Summary linked to this article.

## Supplementary information

Supplementary Information

Description of Additional Supplementary Files

Supplementary Data 1

Supplementary Data 2

Supplementary Data 3

Supplementary Data 4

Supplementary Data 5

Supplementary Movie 1

Supplementary Movie 2

Supplementary Movie 3

Reporting Summary

## Data Availability

Unique materials are available to others and can be obtained by contacting the corresponding author. Patient-derived GCGR cell lines are available via the glioma cellular genetics resource: (www.gcgr.org.uk). The raw reads sequencing data and unprocessed counts have been deposited in GEO (GSE139261), processed sequencing count data are available in Supplementary data 1–5. Data for all figures can be found in manuscript, in Supplementary Figures and Supplementary Tables, or from the corresponding author upon reasonable request. Source data are provided with this paper.

## Source data

Source Data
