# Author Correction: The white matter is a pro-differentiative niche for glioblastoma

**DOI:** 10.1038/s41467-022-29916-y

**Published:** 2022-04-14

**Authors:** Lucy J. Brooks, Melanie P. Clements, Jemima J. Burden, Daniela Kocher, Luca Richards, Sara Castro Devesa, Leila Zakka, Megan Woodberry, Michael Ellis, Zane Jaunmuktane, Sebastian Brandner, Gillian Morrison, Steven M. Pollard, Peter B. Dirks, Samuel Marguerat, Simona Parrinello

**Affiliations:** 1 Samantha Dickson Brain Cancer Unit, UCL Cancer Institute, London, WC1E 6DD UK; 2 MRC Laboratory for Molecular Cell Biology, University College London, Gower Street, London, WC1E 6BT UK; 3 Division of Neuropathology, National Hospital for Neurology and Neurosurgery, University College London NHS Foundation Trust, Queen Square, WC1N 3BG London, UK; 4 Department of Neurodegenerative Disease, UCL Institute of Neurology, Queen Square, WC1N 3BG London, UK; 5 MRC Centre for Regenerative Medicine and Edinburgh Cancer Research UK Cancer Centre, University of Edinburgh, 5 Little France Drive, Edinburgh, EH16 4UU UK; 6 Division of Neurosurgery, Arthur and Sonia Labatt Brain Tumor Research Center, Departments of Surgery and Molecular Genetics, Hospital for Sick Children, Toronto, ON M5G 1X8 Canada; 7 MRC London Institute of Medical Sciences, Du Cane Road, London, W12 0NN UK; 8 Institute of Clinical Sciences, Faculty of Medicine, Imperial College London, Du Cane Road, London, W12 0NN UK

**Keywords:** Cancer microenvironment, Cancer stem cells, CNS cancer

Correction to: *Nature Communications* 10.1038/s41467-021-22225-w, published online 12 April 2021.

In this article the funding from ‘The Oli Hilsdon Foundation’ was omitted. The original article has been corrected.

