# Supplementary material for: The white matter is a pro-differentiative niche for glioblastoma
Source: Nat Commun. 2021 Apr 12;12:2184. doi: 10.1038/s41467-021-22225-w (PMC8042097; doi:10.1038/s41467-021-22225-w)
Supplement: Supplementary file 1 — Supplementary Information [file 41467_2021_22225_MOESM1_ESM.pdf]

Brooks et al., Supplementary Fig. 1

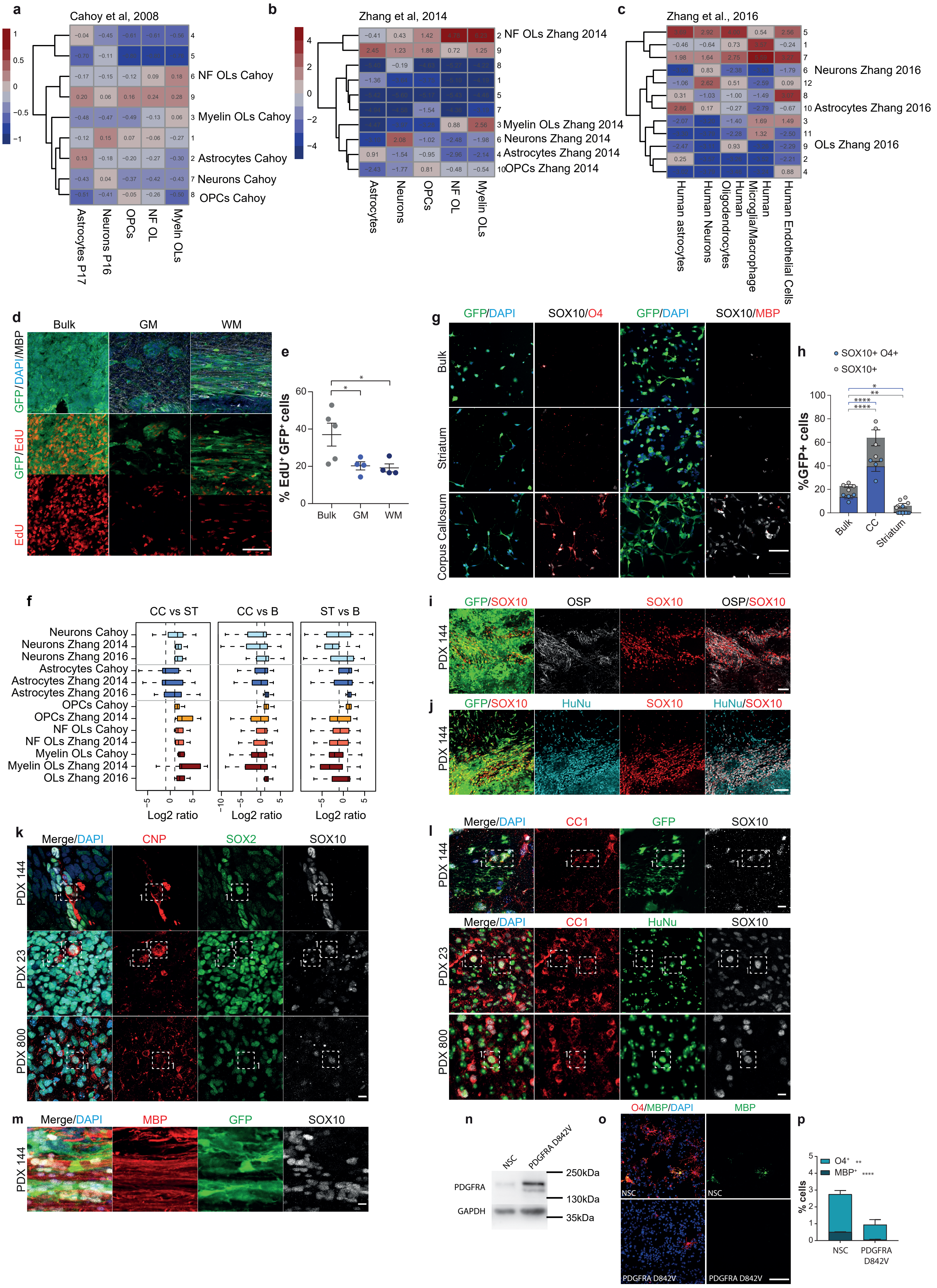

Supplementary Fig. 1: Derivation of brain cell signatures.

a-c, K-means clustering analysis of median centred expression scores for genes highly variable in published cell-type specific mouse/human brain transcriptomes. Specific clusters were used to define cell type signatures indicated on the figure (Supplementary Table 3). **d**, Representative immunofluorescence images and **e**, quantifications of EdU incorporation (red) in GFP^{+} tumour cells within bulk, grey matter (GM), white matter (WM) in G144 xenografts (PDX144). ≥200 cells counted/xenograft. Mean±SEM, n=3-4 xenografts. GM p=0.04, WM p=0.03. Scale=100µm, Two-way ANOVA with Sidak's multiple comparisons test. **f**, Boxplots of DEseq2 expression ratio for indicated gene signatures in corpus callosum (CC) vs striatum (ST, left), CC vs bulk (B, middle) and ST vs B (right) comparisons. Boxplots represent median, interquartile range, and most extreme data points that are not more than 1.5 times the interquartile range. **g**, representative images and **h**, quantifications of GFP^{+} tumour cells isolated from indicated regions, acutely cultured and stained for SOX10 (grey), pre-oligodendrocyte marker O4 (red) or myelinating oligodendrocyte marker MBP (red). ≥140 cells/xenograft counted. Mean±SEM, n=6 xenografts. $SOX10^{+}$: B v. CC p<0.0001, B v. ST p=0.002, $SOX10^{+}/O4^{+}$ B v. CC p<0.0001, B v. ST p=0.03. Scale=100µm, Two-way ANOVA with Sidak's multiple comparisons test. **i**, high magnification images of SOX10 (grey), OSP (red) immunofluorescence of GFP-labelled PDX144 tumours. Note disrupted OSP^{+} myelin fibres. Scale=100µm. **j**, high magnification images of human nuclear antigen (HuNu,turquoise), SOX10 (red), DAPI (blue) immunofluorescence of PDX144 tumours. Scale=100µm. **k-m**, immunofluorescence for the immature oligodendrocyte markers CNP (k, red), CC1 (l, red), and mature marker MBP (m, red) in indicated xenografts. SOX2, GFP, HuNu (green) were used to identify tumour cells as indicated. Dashed square boxes highlight examples of marker positive tumour cells. Scale=10µm. **n**, western blot analysis of PDGFRA in control and PDGFRA-overexpressing NSCs used in o and p confirming transduction efficacy. loading control=GAPDH.

o, immunofluorescence of control or constitutively active PDGFRA (PDGFRA D842V) over-expressing neural stem cells (NSC) cultured 7 days in differentiation media and stained for O4 (red) and MBP (green). Scale=100µm. **p**, quantifications of $O4^{+}$ pre-oligodendrocytes and MBP^{+} mature oligodendrocytes in the cultures shown in n. ≥3500 cells counted across duplicate coverslips/biological repeat. Mean±SEM, n=4 cultures, O4 $p=0.003$, MBP $p<0.0001$. Unpaired two-tailed Student's t test.

Brooks et al., Supplementary Fig. 2

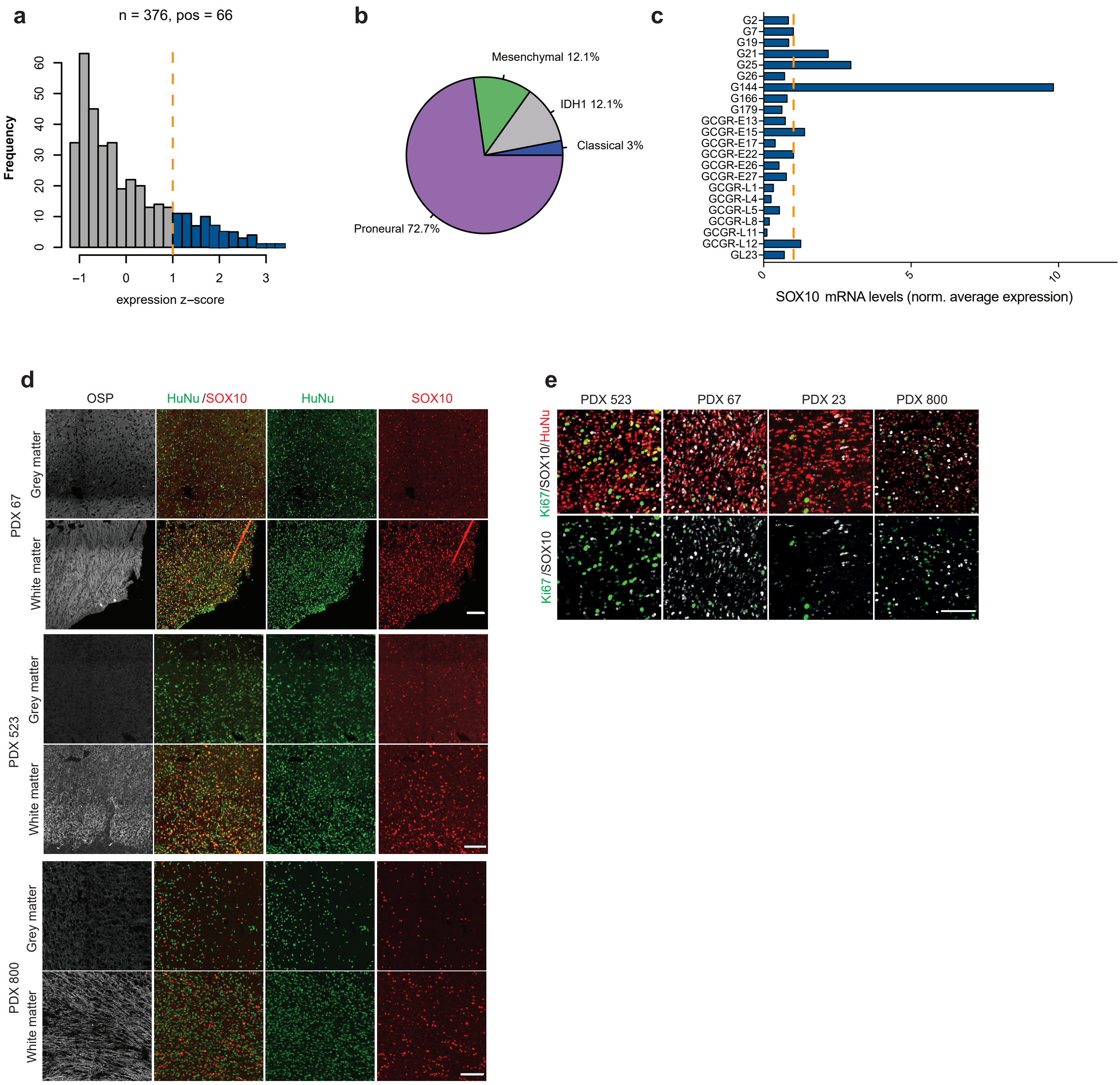

Supplementary Fig. 2: SOX10 expression in TCGA datasets and a panel of patient-derived xenografts.

a, frequency distribution of SOX10 mRNA expression z-scores in 376 GBM tumours from the TGCA database. Dashed line marks the z-score = 1 cut off used in this study. Sixty-six tumours (17.6%) have SOX10 expression above cut off (blue). **b**, proportion of IDH1wt GBM subtypes and IDH1mut GBMs among the 66 SOX10 positive tumours from a. **c**, RT-qPCR analysis of SOX10 mRNA levels in a panel of GSC lines. Dotted line indicates mean expression. **d,** representative immunofluorescence images of $SOX10^+$ patient-derived xenografts quantified in Fig. 2b (PDX67, 523, 800) stained for SOX10 (red), OSP (grey) and human nuclear antigen (HuNu, green). Scale=100µm. **e**, SOX10 (grey), HuNu (red) and Ki67 (green) immunofluorescence images of PDX tumours quantified in Fig. 2c.

Brooks et al., Supplementary Fig. 3

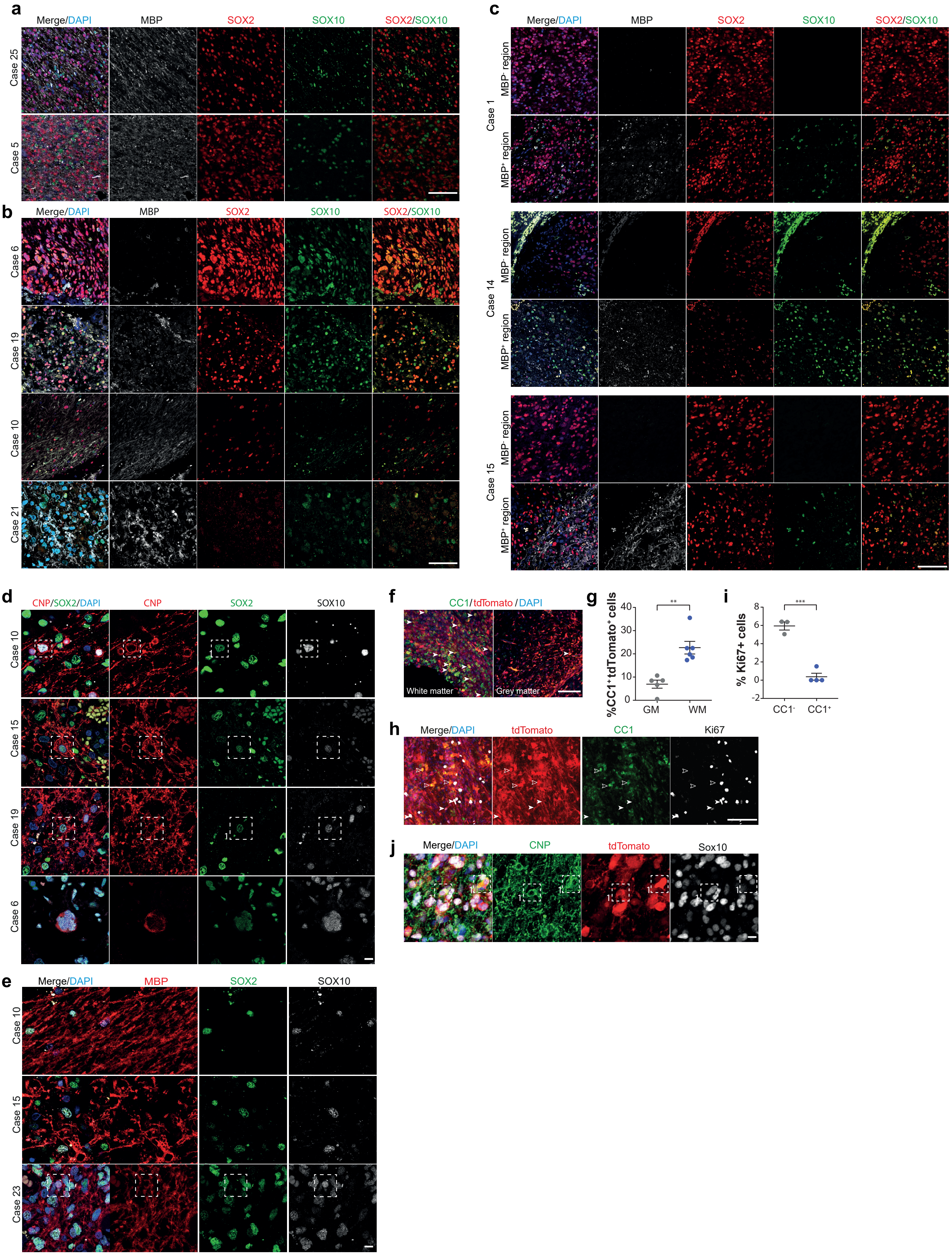

Supplementary Fig. 3: SOX10 expression in primary patient tumours.

a, MBP (grey), SOX10 (green) and SOX2 (red) immunofluorescence staining of white matter regions of $SOX10^-$ patient tumours. No or rare $SOX2^+/SOX10^+$ endogenous OPCs are observed, confirming that SOX2 can be used to identify tumour cells. Scale=100µm. **b,** Representative images of patient tumours stained for SOX2 (red), SOX10 (green), MBP (grey) and DAPI (blue). Scale=100µm. **c**, Representative images of indicated patient tumours stained for SOX2 (red), SOX10 (green), MBP (grey) and DAPI (blue) in tumour areas containing (MBP^+) and devoid of (MBP^-) white matter. Scale=100µm. Note that SOX10 induction is specific to white matter regions. **d**, immunofluorescence staining for the immature oligodendrocyte marker CNP (red) and **e**, the myelinating oligodendrocyte marker MBP (red) in patient material. SOX2 (green) was used to identify tumour cells. Dashed square boxes highlight examples of marker positive tumour cells in d and endogenous oligodendrocytes in e. No MBP^+ tumour cells are observed amongst MBP^+ endogenous oligodendrocytes. Scale=10µm. **f**, representative immunofluorescence image of CC1 (green) staining of a $tdTomato^+$ (red) *Nf1/Pten/p53* mouse tumour. Arrowheads indicate examples of $CC1^+$ $tdTomato^+$ tumour cells. Scale=100µm. **g**, quantification of the percentage of differentiated $CC1^+$ tumour cells in white (WM) and grey matter (GM). For each tumour, ≥700 cells were quantified across 2 independent ROIs selected within white or grey matter. Mean±SEM, n=3 tumours, p=0.001, unpaired two-tailed Student's t test. **h**, representative immunofluorescence images of $tdTomato^+$ *Nf1/ Pten/p53* mouse tumours stained for CC1 (green) and Ki67 (grey). Open arrowheads denote $CC1^+/Ki67^-$ and closed arrowheads $CC1^-/Ki67^+$ tumour cells. Scale=100µm. **i**, quantification of percentage of proliferating ($Ki67^+$) $CC1^+$ and $CC1^-$ tumour cells in the stainings shown in h. For each tumour, ≥400 cells were quantified across 2 independent ROIs. Mean±SEM, n=3 tumours. p=0.0002, unpaired two-tailed Student's t test. **j**, representative immunofluorescence image of a tdTomato+ (red) Nf1/Pten/p53 mouse tumour stained for CNP (green) and Sox10 (grey). Scale=10µm.

Brooks et al., Supplementary Fig. 4

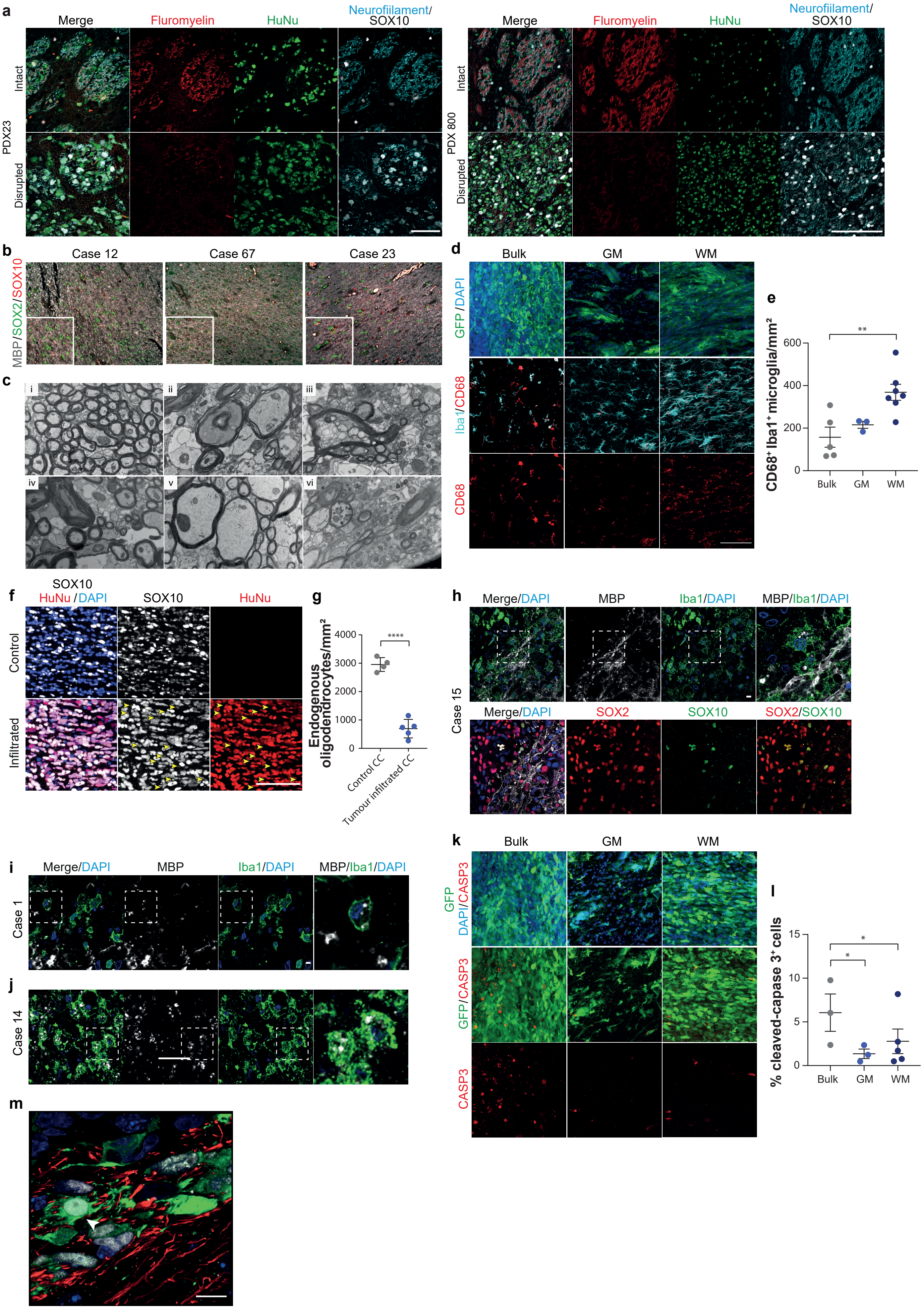

Supplementary Fig. 4: SOX10 upregulation does not occur in intact myelin.

a, Fluoromyelin (red), SOX10 (grey), Neurofilament (turquoise), human nuclear antigen (HuNu, green) staining of white matter regions of xenografts quantified in Fig. 2b. Scale=100µm. **b,** Representative images of patient tumours stained for SOX2 (green), SOX10 (red), MBP (grey), DAPI (blue). Note the majority of $SOX2^{+}$ tumour cells do not express SOX10 in regions of more intact myelin. Scale=50µm. **c**, EM micrographs depicting examples of axonal degeneration and myelin disruption phenotypes observed in tumour-infiltrated white matter: *i* intact contralateral axons, *ii* dark axon, *iii* vacuolised axon, *iv* axon with condensed axoplasm, *v* swollen axon, *vi* axon with enlarged organelles. Scale=1µm. **d**, Representative immunofluorescence and **e**, quantifications of $Iba1^{+}$ (turquoise)/$CD68^{+}$ (red) activated microglia in the bulk, grey matter (GM) and white matter (WM) of GFP^{+} G144 xenografts (PDX144). ≥140 cells/region per xenograft were counted. Mean±SEM, n=3-4 xenografts. p=0.005. Scale=100µm, Two-way ANOVA with Sidak's multiple comparisons test. **f**, representative immunofluorescence of the corpus callosum invaded by tumour cells in a terminal PDX144 stained for SOX10 (grey), HuNu (red), DAPI (blue). Scale=50µm. **g**. quantification of $SOX10^{+}$/$HuNu^{-}$ endogenous oligodendroglia in the images in f. ≥450 cells counted across 2 ROIs per xenograft. Mean ±SEM, n= 2 normal brains, n=5 xenografts p<0.0001. Unpaired two-tailed Student's t test. **h-j**, immunofluorescence of $Iba1^{+}$ microglia (green) with engulfed MBP myelin debris (grey, dashed box) in tumour infiltrated white matter of patient tumours. For case 15 in h the sequential section was stained for SOX2 (red) and SOX10 (green), confirming that tumour cell differentiation occurs in areas of demyelination (bottom panels). Scale=10µm. **k**, Representative immunofluorescence and **l**, quantifications of cleaved $Caspase^{+}$ (red) staining of GFP^{+} (green) tumour cells in the bulk, GM and WM regions of GFP-labelled PDX144 xenografts. ≥200 cells counted per region per xenograft. Scale=100µm Mean±SEM, n=3 xenografts. No significant differences in apoptosis were found. Two-way ANOVA with Sidak's multiple comparisons

test. **m**, representative super-resolution image of a demyelinating region within a PDX144. Sections were stained for SOX10 (grey), neurofilament (NF, red). Endogenous GFP fluorescence is in green and nuclei are counterstained with DAPI (blue). Arrowhead indicates a pre-oligodendrocyte tumour cell with multiple cellular processes aligned to axons. Scale=10μm.

Brooks et al., Supplementary Fig. 5

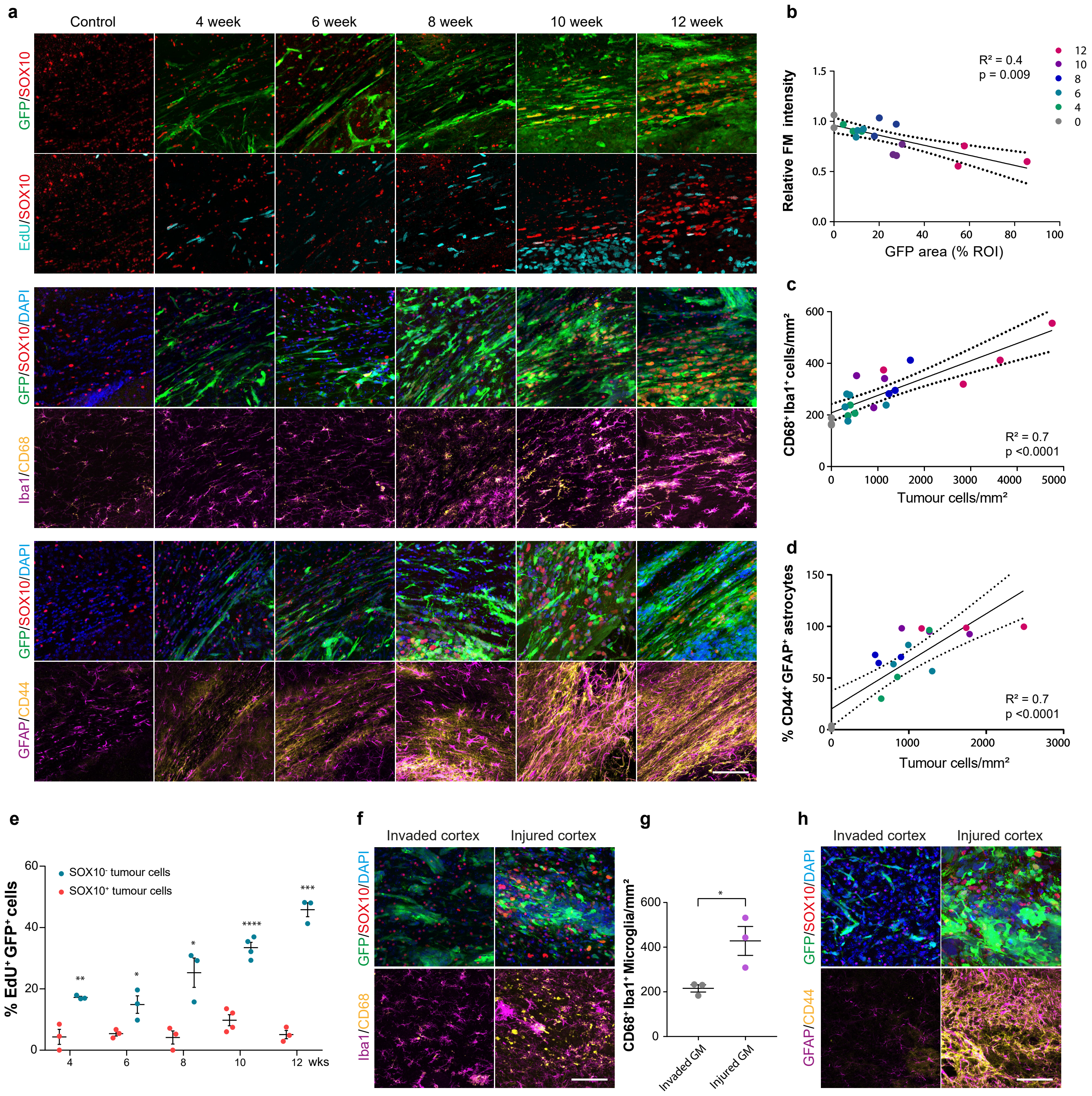

Supplementary Fig. 5: GBM differentiation is an injury response.

a, time-course analysis of GBM cell infiltration into the corpus callosum in GFP-labelled G144 xenografts (PDX144) collected at indicated time-points (weeks). Representative immunofluorescence images of each time point are shown. All samples were stained for SOX10 (red) and GFP (green) to visualise extent of infiltration and tumour cell differentiation (top panels). In addition, top samples were stained for SOX10 (red) and EdU (turquoise) to assess stability of differentiation, middle samples for Iba1 (magenta) and CD68 (yellow) to assess microglia activation and lower panels for GFAP (magenta) and CD44 (yellow) to examine reactive astrocytes**.** Scale=100µm. **b**, quantification of the number of endogenous oligodendrocytes, **c**, activated microglia and **d**, reactive astrocytes in the corpus callosum as a function of number of invaded tumour cells. Dots indicate mean cell density in individual xenografts colour-coded by time. A minimum ROI area of 300µm was analysed. The coefficient of determination (R^2) and p value are indicated. **e**, quantification of the percentage of proliferative (EdU^+) $SOX10^+$ and $SOX10^-$ GFP^+ G144 cells in the time-course experiments shown in a. Note that the percentage of $SOX10^+/EdU^+$ remained constant indicative of stable differentiation. A minimum ROI area of 300µm was analysed. Mean±SEM, n=3 xenografts per time point. 4wk p=0.007, 6wk p=0.03, 8wk p=0.02, 10wk p=0.00008, 12wk p=0.0001. Multiple t-tests using the Holm-Sidak method. **f**, representative immunofluorescence images and **g**, quantifications of SOX10 (red), Iba1 (magenta), CD68 (yellow) and DAPI (blue) immunofluorescence staining of

GFP^+ G144 cells directly injected (injured CTX) or invaded into the dense myelin of inner cortex from the tumour bulk (invaded CTX). Scale=100µm. ≥90 cells per xenograft were counted. Mean±SEM, n=3 xenografts per group. p=0.03. Unpaired two-tailed Student's t test. **h**, SOX10 (red), GFAP (magenta), CD44 (yellow) and DAPI (blue) immunofluorescence staining of GFP^+ G144 cells directly injected (injured CTX) or invaded into the dense myelin of inner

cortex from the tumour bulk (invaded CTX). Note that injection into the inner cortex causes glial activation consistent with an injury response. Scale=100µm.

Brooks et al., Supplementary Fig. 6

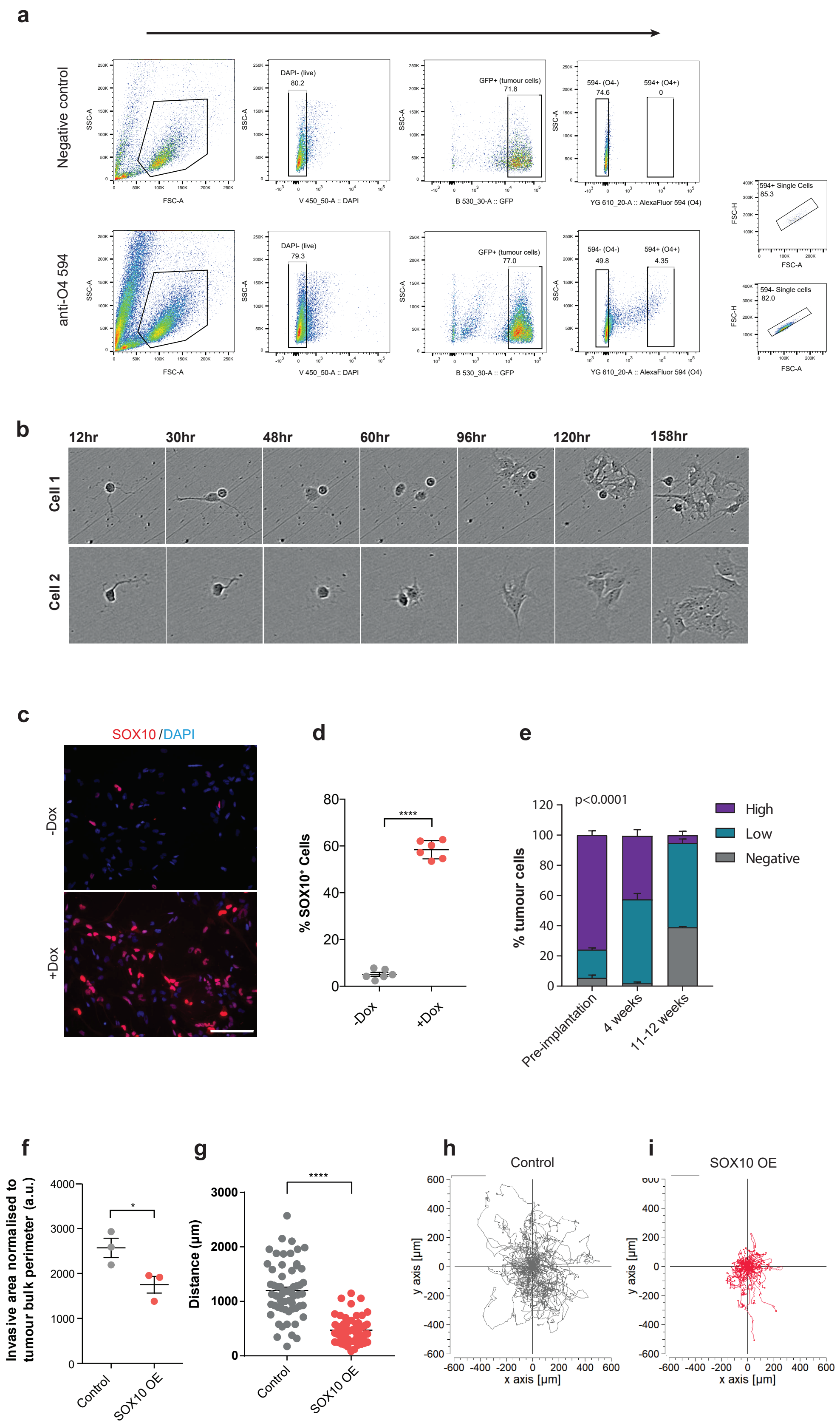

Supplementary Figure 6: SOX10 up-regulation reduces tumour cell motility.

a, representative FACS plots of the purification of $O4^+/GFP^+$ G144 cells from corpus callosum (CC) and bulk (B) regions of xenografts related to b in this figure and Fig. 6g. Unstained GFP^+ G144 cells from the same regions were used for gating. **b**, representative phase contrast still images taken from videos of $O4^+$ G144 cells acutely FACS-sorted from the B and CC regions of primary xenografts and seeded in neural stem cell conditions for 7 days. **c**, representative images of SOX10 (red) and DAPI (blue) fluorescence staining of G144 cultures before (Dox^-) and after (Dox+) SOX10 induction with doxycycline for 48h. Scale=100µm. **d**, quantification of percentage of SOX10 expressing cells in cultures from c. ≥90 cells were counted per group. Mean±SEM, n=2 independent cultures each on triplicate coverslips. $p<0.0001$. Unpaired Student's t-test. **e**, quantification of percentages of $SOX10^-$, SOX10low and SOX10high tumour cells in SOX10-overespressing (SOX10 OE) G144 xenografts. Quantifications were done before tumour implantation *in vitro* (pre-implantation) or at 4 or 11-12 weeks post-implantation in grey matter regions of xenografs, as indicated. ≥250 cells per group were counted. Mean ±SEM, n=3 cultures or xenografts per group. $p<0.0001$. Two-way ANOVA. **f**, quantification of area occupied by invasive tumour cells in Control and SOX10 OE tumours shown in Fig. 6e. Quantifications were performed across 2 sections per mouse. Mean ±SEM, n=3 mice/group. $p=0.04$. Unpaired two-tailed Student's t test. **g**, quantification of migrated distance of individual G144 cells in Control and SOX10 OE G144 cultures. Each dot represents a cell. Mean±SEM, n=60 cells per group pooled from 3 independent transductions. $p<0.0001$. Unpaired Student's t-test. **h-i**, traces of individual migrating cells from a, aligned to the same point of origin.

Brooks et al., Supplementary Fig. 7

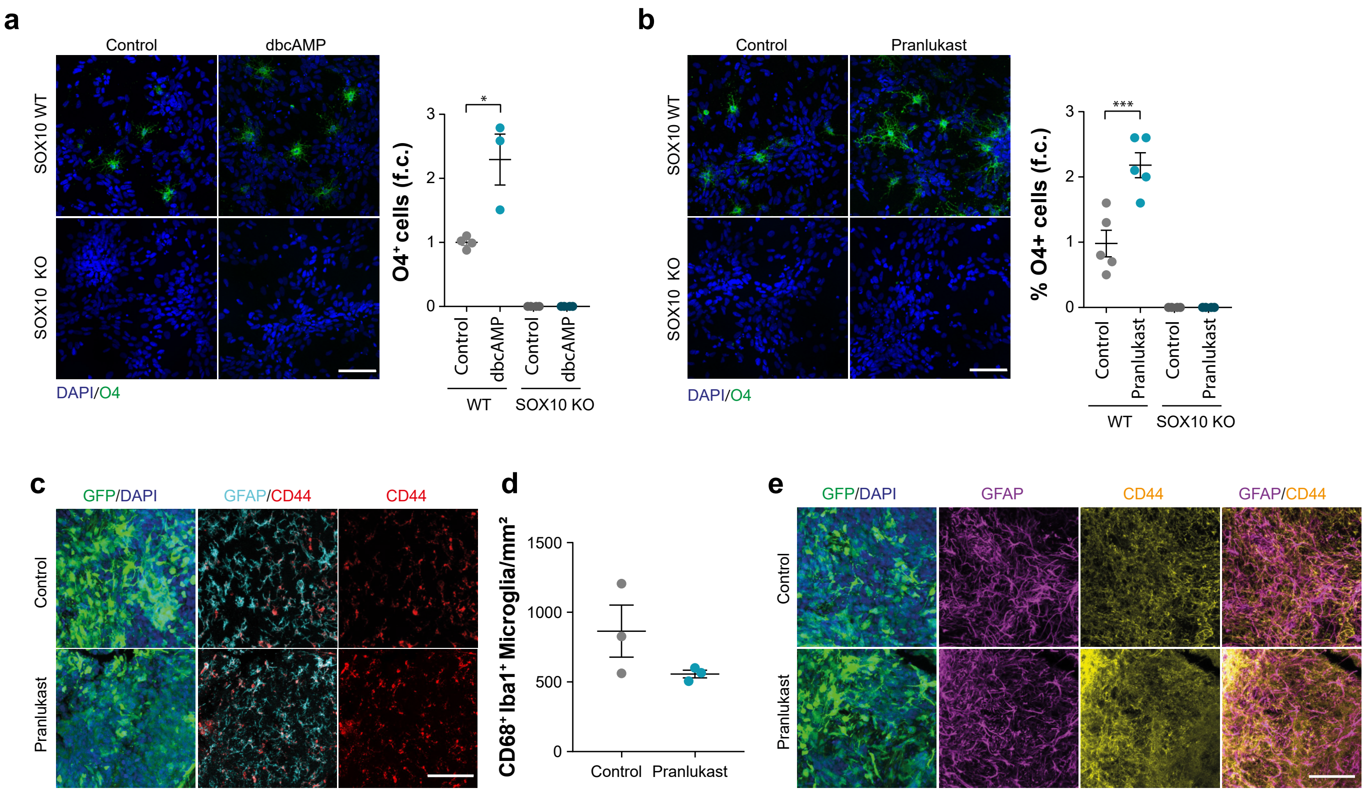

Supplementary Fig. 7: Myelination-promoting drugs induce GBM differentiation via SOX10.

a, representative immunofluorescence images (left) of control and SOX10 knock-out G144 cultures untreated or treated with db-cAMP or b, Pranlukast for 14 days in the absence of growth factors and stained for O4+ (green). Quantifications of the percentage of O4+ cells in the same cultures are shown on the right. ≥1000 cells cells across duplicate coverslips were counted per biological repeat. Mean±SEM, n=3-5 independent cultures per group, a, $p=0.01$, b, $p=0.003$. Unpaired two-tailed Student's t test. c, representative immunofluorescence images and d, quantifications of Iba1+ (turquoise) and CD68+ (red) activated microglia staining in GFP+ PDX144 treated with saline or pranlukast Scale=100µm. ≥400 cells per xenograft were counted. Mean±SEM, n=3 xenografts per group. *$p<0.05$. Unpaired two-tailed Student's t test. e, GFAP (magenta), CD44 (yellow) and DAPI (blue) immunofluorescence staining of activated astrocytes in GFP+ G144 tumours treated with saline or pranlukast. Note that Pranlukast did not affect endogenous glia. Scale=100µm.

Supplementary table 1: Description of RNA-seq datasets

Exp	Name	ID	Description	Mouse	Passage	Region	Fastq R1	Fastq R1	Fastq R2
In vivo	B.1	M1_1	Mouse 1 Bulk	1	.	Bulk	M1_1_TAAGGCGA-CTCTCTAT_L001_R1_001.fastq	M1_1_TAAGGCGA-CTCTCTAT_L002_R1_001.fastq	.
	CC.1	M1_2	Mouse 1 CC	1	.	Corpus Callosum	M1_2_CGTACTAG-CTCTCTAT_L001_R1_001.fastq	M1_2_CGTACTAG-CTCTCTAT_L002_R1_001.fastq	.
	B.2	M2_1	Mouse 2 Bulk	2	.	Bulk	M2_1_GGACTCCT-CTCTCTAT_L001_R1_001.fastq	M2_1_GGACTCCT-CTCTCTAT_L002_R1_001.fastq	.
	CC.2	M2_2	Mouse 2 CC	2	.	Corpus Callosum	M2_2_TAGGCATG-CTCTCTAT_L001_R1_001.fastq	M2_2_TAGGCATG-CTCTCTAT_L002_R1_001.fastq	.
	ST.2	M2_4	Mouse 2 Striatum	2	.	Striatum	M2_4_CAGAGAGG-CTCTCTAT_L001_R1_001.fastq	M2_4_CAGAGAGG-CTCTCTAT_L002_R1_001.fastq	.
	ST.3	M3_4	Mouse 3 Striatum	3	.	Striatum	M3_4_TCCTGAGC-TATCCTCT_L001_R1_001.fastq	M3_4_TCCTGAGC-TATCCTCT_L002_R1_001.fastq	.
	B.4	M4_1	Mouse 4 Bulk	4	.	Bulk	M4_1_GGACTCCT-TATCCTCT_L001_R1_001.fastq	M4_1_GGACTCCT-TATCCTCT_L002_R1_001.fastq	.
	CC.4	M4_2	Mouse 4 CC	4	.	Corpus Callosum	M4_2_TAGGCATG-TATCCTCT_L001_R1_001.fastq	M4_2_TAGGCATG-TATCCTCT_L002_R1_001.fastq	.
	ST.4	M4_4	Mouse 4 Striatum	4	.	Striatum	M4_4_CAGAGAGG-TATCCTCT_L001_R1_001.fastq	M4_4_CAGAGAGG-TATCCTCT_L002_R1_001.fastq	.
In vitro	G144_p34_Ctl	G144_p34_Ctl	G144 Tet-ON SOX10	.	34	.	G144_p34_Ctl_S73_L006_R1_001.fastq	.	G144_p34_Ctl_S73_L006_R2_001.fastq
	G144_p34_Dox	G144_p34_Dox	G144 Tet-ON SOX10 + Doxycyclin	.	34	.	G144_p34_Dox_S72_L006_R1_001.fastq	.	G144_p34_Dox_S72_L006_R2_001.fastq
	G144_p35_Ctl	G144_p35_Ctl	G144 Tet-ON SOX10	.	35	.	G144_p35_Ctl_S75_L006_R1_001.fastq	.	G144_p35_Ctl_S75_L006_R2_001.fastq
	G144_p35_Dox	G144_p35_Dox	G144 Tet-ON SOX10 + Doxycyclin	.	35	.	G144_p35_Dox_S74_L006_R1_001.fastq	.	G144_p35_Dox_S74_L006_R2_001.fastq
	G144_p36_Ctl	G144_p36_Ctl	G144 Tet-ON SOX10	.	36	.	G144_p36_Ctl_S77_L006_R1_001.fastq	.	G144_p36_Ctl_S77_L006_R2_001.fastq
	G144_p36_Dox	G144_p36_Dox	G144 Tet-ON SOX10 + Doxycyclin	.	36	.	G144_p36_Dox_S76_L006_R1_001.fastq	.	G144_p36_Dox_S76_L006_R2_001.fastq

#Exp: experiment type, "In vivo" relates to figure 1, "In vitro" to figure 3.
#Name: unique dataset name
#ID: unique dataset ID
#Description: sample description
#Mouse: animal unique ID
#Passage: Cell line passage
#Region: location of tumour cells in vivo
#Fastq R1: Raw RNA-seq files as in GEO submission (Read 1)
#Fastq R2: Raw RNA-seq files as in GEO submission (Read 2)

Supplementary table 2: Molecular characteristics of GSC lines used in this study

GSC line	TCGA Subtype	Heidelberg classifier
G7	P	N/A
G21	P/C	N/A
G25	M	N/A
G26	P/C	N/A
G144	P	N/A
G166	M	N/A
G179	M	N/A
GCGR-E13	C	RTKII
GCGR-E15	N/A	RTKII
GCGR-E17	C	RTKII
GCGR-E27	N/A	RTKI
GL23	N/A	N/A

P: Proneural
C: Classical
M: mesenchymal

Supplementary table 3: Patient information for tumours used for SOX10 immunofluorescence

IDH1 WT	Age	Gender	Location	Laterality	Path diagnosis	EGFR	SOX10
Case 23	48	M	Temporal	Left	GBM, IDH-wildtype	No amplification	+++
Case 67	60	F	Temporal	Right	GBM, IDH-wildtype	Ampliffication	++
Case 12	65	M	Frontal	Left	GBM, IDH-wildtype	No amplification	+++
Case 1	56	M	Temporal	Left	GBM, IDH-wildtype	Ampliffication	+
Case 3	47	M	Parietal	Left	GBM, IDH-wildtype	Ampliffication	++
Case 4	47	M	Frontal	Right	GBM, IDH-wildtype	Ampliffication	+
Case 5	44	M	Temoral and Parie	Right	GBM, IDH-wildtype	Ampliffication	-
Case 6	32	M	Temporal	Left	GBM, IDH-mutant	No amplification	+++
Case 8	55	M	Frontal	Right	GBM	Not available	-
Case 9	50	M	Frontal	Right	GBM, IDH-mutant	No amplification	-
Case 10	27	M	Temporal	Left	Astrocytoma, IDH-mutant	No amplification	+
Case 11	61	M	Parietal-Occipital	Left	GBM	Not available	+
Case 13	63	M	Temporal	Left	GBM	Not available	-
Case 14	47	M	Frontal	Bilateral	GBM	Not available	+
Case 15	50	M	Frontal	Right	GBM, IDH-wildtype	No amplification	++
Case 16	41	F	Temporal	Right	GBM, IDH-wildtype	No amplification	-
Case 17	44	M	Temporal	Right	GBM	Not available	+
Case 18	40	F	Temporal	Left	GBM, IDH-wildtype	No amplification	+
Case 19	55	M	Temporal and Occipital	Right	GBM	Not available	+++
Case 21	55	M	Frontal	Left	GBM	Not available	++
Case 22	41	F	Frontal	Right	GBM	Not available	+
Case 24	55	F	Tempo	NA	GBM	Not available	+
Case 25	45	F	Frontal	Left	GBM, IDH-mutant	No amplification	-
Case 26	42	M	Frontal	Right	GBM, IDH-mutant	No amplification	-
Case 27	67	F	Frontal and Parietal	Left	GBM, IDH-wildtype	Ampliffication	-
Case 28	42	M	Temporal	Right	GBM, IDH-wildtype	No amplification	++

#IDH1 WT: patient ID
#Age: patient age at time of prelevement.
#Gender: patient gender.
#Location: tumour bulk location.
#Laterality: tumour location relative to the fronto-occipital axis.
#Path diagnosis: pathology diagnostic.
#EGFR: EGFR gene genomic copy number amplification.

Supplementary table 4: Primer squences

GAPDH F	CCTCACAGATCGCCTACACC
GAPDH R	CATATAGGAGAAGGCCGAGTAGA
SOX10 F	GTCTCCTCTGACTTCAACAGCG
SOX10 R	ACCACCCTGTTGCTGTAGCCAA
