# Supplementary material for: The white matter is a pro-differentiative niche for glioblastoma
Source: Nat Commun. 2021 Apr 12;12:2184. doi: 10.1038/s41467-021-22225-w (PMC8042097; doi:10.1038/s41467-021-22225-w)
Supplement: Supplementary file 2 — Description of Additional Supplementary Files [file 41467_2021_22225_MOESM2_ESM.pdf]

Description of Additional Supplementary Files

File Name: Supplementary Data 1
Description: DEseq2 analysis of in vivo RNA-seq data.

File Name: Supplementary Data 2
Description: K-means clustering of GBM invasion transcriptomic signatures.

File Name: Supplementary Data 3
Description: Gene expression signatures defined in this study.

File Name: Supplementary Data 4
Description: DEseq2 analysis of in vitro RNA-seq data

File Name: Supplementary Data 5
Description: GO analysis of SOX10-upregualted genes

File Name: Supplementary Movie 1
Description: 3D animation of a representative immunofluorescence image of a GFPlabelled PDX144 stained for CD68 (red) and MBP (grey) showing the presence of microglia with engulfed myelin debris in infiltrated white matter.

File Name: Supplementary Movie 2 & 3
Description: Time-lapse imaging of $O4^+$ tumour cells FAC-sorted from the corpus callosum of G144 tumours and cultured in stem cell media for 7 days. Two examples of cells losing oligodendrocyte morphology and re-entering the cell-cycle are shown.
