# Supplementary material for: The white matter is a pro-differentiative niche for glioblastoma
Source: Nat Commun. 2021 Apr 12;12:2184. doi: 10.1038/s41467-021-22225-w (PMC8042097; doi:10.1038/s41467-021-22225-w)
Supplement: Supplementary file 11 — Reporting Summary [file 41467_2021_22225_MOESM11_ESM.pdf]

nature research

Corresponding author(s): Simona Parrinello

Last updated by author(s): Feb 8, 2021

Reporting Summary

Nature Research wishes to improve the reproducibility of the work that we publish. This form provides structure for consistency and transparency in reporting. For further information on Nature Research policies, see Authors & Referees and the Editorial Policy Checklist.

Statistics

For all statistical analyses, confirm that the following items are present in the figure legend, table legend, main text, or Methods section.

n/a	Confirmed	
☐	☒	The exact sample size (*n*) for each experimental group/condition, given as a discrete number and unit of measurement
☐	☒	A statement on whether measurements were taken from distinct samples or whether the same sample was measured repeatedly
☐	☒	The statistical test(s) used AND whether they are one- or two-sided<br>*Only common tests should be described solely by name; describe more complex techniques in the Methods section.*
☒	☐	A description of all covariates tested
☐	☒	A description of any assumptions or corrections, such as tests of normality and adjustment for multiple comparisons
☐	☒	A full description of the statistical parameters including central tendency (e.g. means) or other basic estimates (e.g. regression coefficient) AND variation (e.g. standard deviation) or associated estimates of uncertainty (e.g. confidence intervals)
☐	☒	For null hypothesis testing, the test statistic (e.g. F, t, r) with confidence intervals, effect sizes, degrees of freedom and P value noted<br>*Give P values as exact values whenever suitable.*
☒	☐	For Bayesian analysis, information on the choice of priors and Markov chain Monte Carlo settings
☒	☐	For hierarchical and complex designs, identification of the appropriate level for tests and full reporting of outcomes
☐	☒	Estimates of effect sizes (e.g. Cohen's *d*, Pearson's *r*), indicating how they were calculated

Our web collection on statistics for biologists contains articles on many of the points above.

Software and code

Policy information about availability of computer code

Data collection	Confocal images were acquired using Zeiss Zen black 2011 v8.1.<br>Sequencing data were collected on a HiSeq 2500 instrument at the MRC LMS genomics facility processed using the RTA software (1.18.64) and reads demultiplexed using CASAVA 1.8.4 or 2.17.<br>Differential expression analysis was performed and normalized counts were generated using the DESeq2 Bioconductor package version 1<br>IVIS images were acquired and quantifications performed using Living Image software 4.3.1.0.15880 (Xenogen, Caliper Life Sciences).<br>FACS DIVA version 8.0.1 was used for FACS of tumour cells<br>FlowJo 10.7.1 was used to illustrate the gating strategy in extended data figure 6a
Data analysis	Statistical tests were conducted using Prism 7.0 (GraphPad) software for all analyses.<br>Sequencing reads were mapped and feature count data extracted using the STAR aligner 2.6 (Dobin et al 2014). GO analysis was performed using the VLAD (http://proto.informatics.jax.org/prototypes/vlad/) . Analysis of RNA-seq data was performed using R-3.6.1. R scripts are available upon request.<br>Confocal microscopy image analysis was performed using Fiji ImageJ v1.52p.

For manuscripts utilizing custom algorithms or software that are central to the research but not yet described in published literature, software must be made available to editors/reviewers. We strongly encourage code deposition in a community repository (e.g. GitHub). See the Nature Research guidelines for submitting code & software for further information.

Data

Policy information about availability of data

All manuscripts must include a data availability statement. This statement should provide the following information, where applicable:

- Accession codes, unique identifiers, or web links for publicly available datasets
- A list of figures that have associated raw data
- A description of any restrictions on data availability

The raw reads sequencing data and unprocessed counts have been deposited in GEO (GSE139261), processed sequencing count data are available in

Supplementary Tables 1 and Supplementary Data 1-5. Data for all figures can be found in manuscript, in Supplementary Data files and Supplementary Tables, or from corresponding author upon reasonable request. Source data are provided with this paper.

Field-specific reporting

Please select the one below that is the best fit for your research. If you are not sure, read the appropriate sections before making your selection.

☒ Life sciences ☐ Behavioural & social sciences ☐ Ecological, evolutionary & environmental sciences

For a reference copy of the document with all sections, see nature.com/documents/nr-reporting-summary-flat.pdf

Life sciences study design

All studies must disclose on these points even when the disclosure is negative.

Sample size	Sample size for all experiments was based on previously published studies and from previous experience using the same models. No statistics were used to predetermine sample size. (Krusche et al. 2016., Lan et al. 2017., Pollard et al. 2009.)
Data exclusions	Mice in which extraparenchymal tumours developed resulting from technical issues or where a cause of death could not be determined were excluded.
Replication	Replicates were used in all experiments as noted in the text. All experiments were repeated at least three times with reproducible results.
Randomization	The nature of the experiments carried out in this study did not require randomisation.
Blinding	Blinding was not possible due to the pronounced and recognisable SOX10-dependant phenotypes. Quantifications were automated as much as possible to remove operator bias.

Reporting for specific materials, systems and methods

We require information from authors about some types of materials, experimental systems and methods used in many studies. Here, indicate whether each material, system or method listed is relevant to your study. If you are not sure if a list item applies to your research, read the appropriate section before selecting a response.

Materials & experimental systems

n/a	Involved in the study	
☐	☒	Antibodies
☐	☒	Eukaryotic cell lines
☒	☐	Palaeontology
☐	☒	Animals and other organisms
☐	☒	Human research participants
☒	☐	Clinical data

Methods

n/a	Involved in the study	
☒	☐	ChIP-seq
☐	☒	Flow cytometry
☒	☐	MRI-based neuroimaging

Antibodies

Antibodies used

Primary antibodies used were mouse anti-CC1 (1:1000, abcam ab16794), rabbit anti-Ki67 (1:250, abcam ab16667), goat anti-MBP (1:1000, santa cruz sc-13912), rat anti-MBP (1:500 paraffin; 1:1000 coverslips, sigma MAB386), chicken anti-neurofilament (1:2000, abcam ab4680), mouse anti-HuNu (1:250, clone 235-1, sigma MAB1281), mouse anti-O4 (1:500, R&D MAB1326), rabbit anti-RFP (1:500, antibodies online AA234 (ABIN129578)), rabbit anti-OSP (1:500, abcam ab53041), goat anti-SOX10 (1:1000, R&D AF2864), rat anti-CD68 (1:500, abcam ab53444), rabbit anti-Iba1 (1:2000, alpha laboratories 019-19741), mouse anti-Sox2 (1:100, abcam ab79351).

Secondary antibodies (Invitrogen): Donkey anti-goat, Alexa fluor 488 (A-11055), Donkey anti-goat, Alexa fluor 555 (A-21432), Donkey anti-goat, Alexa fluor 594 (A-11058), Donkey anti-goat, Alexa fluor 647 (A-21447), Donkey anti-rabbit, Alexa fluor 488 (A-21206), Donkey anti-rabbit, Alexa fluor 555 (A-31572), Donkey anti-rabbit, Alexa fluor 594 (A-21207), Donkey anti-rabbit, Alexa fluor 647 (A32795), Donkey anti-mouse, Alexa fluor 488 (A-21202), Donkey anti-mouse, Alexa fluor 555 (A-31570), Donkey anti-mouse, Alexa fluor 647 (A-31571), Donkey anti-rat, Alexa fluor 594 (A-21209), goat anti-chicken 647 (A21449).

Secondary antibodies (other sources): Donkey anti-chicken 488 (Merck, SAB46000321).

Validation

All antibodies have been validated in the literature and/or had validation data supplied by the manufacturer. Further validation was performed to confirm that each antibody produced the expected cellular patterns and broad distributions.

mouse anti-CC1 (abcam ab16794) - cytoplasmic localisation (>65 citations)

rabbit anti-Ki67 (abcam ab16667) - Knockout validated in Hela cells (>1300 citations)

goat anti-MBP (santa cruz sc-13912) - subcellular location: Myelin membrane; Peripheral membrane protein; Cytoplasmic side (>10 citations).

rat anti-MBP (sigma MAB386) - subcellular locallisation: Myelin membrane; Peripheral membrane protein; Cytoplasmic side. (>190 citations).

chicken anti-neurofilament (abcam ab4680) - subcellular localisation: neuronal cytoplasm (>70 citations)

mouse anti-HuNu (clone 235-1, sigma MAB1281) - nuclear localisation restricted to human cells. It shows no reactivity against mouse (>295 citations).

mouse anti-O4 (R&D MAB1326) - Oligodendrocyte Marker O4 is an antigen on the surface of oligodendrocyte progenitors (1, 2). It has been commonly used as the earliest recognized marker specific for the oligodendroglial lineage (3-8).
1. Schachner, M. et al. (1981) Dev. Biol. 83:328.
2. Bansal, R. et al. (1989) J. Neurosci. Res. 24:548.
3. Bansal, R. and Pfeiffer, S.E. (1989) Proc. Natl. Acad. Sci. USA 86:6181.
4. Gard, A. et al. (1995) Dev. Biol. 167:596.
5. Reynolds, R. and Hardy, R. (1997) J. Neurosci. Res. 47:455.
6. Ono, K. et al. (1997) J. Neurosci. Res. 48:212.
7. Pang, Y. et al. (2000) J. Neurosci. Res. 62:510.
8. Cai, Z. et al. (2001) Brain Res. 898:126.

rabbit anti-RFP (antibodies online AA234 (ABIN129578)) - independently validated by antibodies online (>300 citations)

rabbit anti-OSP (abcam ab53041) - membrane localisation (>50 citations)

goat anti-SOX10 (R&D AF2864) - nuclear localisation. Validated in SOX10 overexpression (figure 5d-e) and knockout (figure 5h-i) experiments (>50 citations)

rat anti-CD68 (abcam ab53444) - localisation to Iba1+ microglia (>170 citations)

rabbit anti-Iba1 (alpha laboratories 019-19741) - (>340 citaitons)

mouse anti-Sox2 (abcam ab79351) - nuclear localisation (>40 citations)

Eukaryotic cell lines

Policy information about cell lines

Cell line source(s)	Primary cell lines were all derived from GBM tumours as previously reported (Pollard et al 2009, Lan et al 2017).<br>293T cells (ATCC) were used only for virus production.
Authentication	Cell lines were confirmed to match their parental primary GBM tumour tissue by microsatellite genotyping and epic array
Mycoplasma contamination	All cell cultures are routinely tested for mycoplasma contamination and all cultures used tested negative.
Commonly misidentified lines<br>(See ICLAC register)	No commonly misidentified lines were used.

Animals and other organisms

Policy information about studies involving animals; ARRIVE guidelines recommended for reporting animal research

Laboratory animals	Female CD1 Nude and NOD-SCID-IL2R gamma chain-deficient (NSG) mice were obtained from Charles River. C57BL6 mice were bred in house. All animals were housed at ambient temperature (22+/- 2C) and 55+/- 10% humidity with lights-on and lights-off times of 12hrs starting at 7am and 7pm respectively.
Wild animals	No wild animals were used
Field-collected samples	No field-collected samples were used.
Ethics oversight	All procedures were performed in compliance with the Animal Scientific Procedures Act, 1986 and approved by with the UCL Animal Welfare and Ethical Review Body (AWERB) in accordance with the International guidelines of the Home Office (UK) or The Hospital for Sick Children's Animal Care Committee.

Note that full information on the approval of the study protocol must also be provided in the manuscript.

Human research participants

Policy information about studies involving human research participants

Population characteristics	The patient cohort consisted of 19 males and 7 females with an average age of 49+/-10years.
Recruitment	Retrospective study, no recruitment.
Ethics oversight	Ethics Committee references 08-077, 115/ES/0094, 08/H0716/16.

Note that full information on the approval of the study protocol must also be provided in the manuscript.

Flow Cytometry

Plots

Confirm that:

☒ The axis labels state the marker and fluorochrome used (e.g. CD4-FITC).

☒ The axis scales are clearly visible. Include numbers along axes only for bottom left plot of group (a 'group' is an analysis of identical markers).

☒ All plots are contour plots with outliers or pseudocolor plots.

☒ A numerical value for number of cells or percentage (with statistics) is provided.

Methodology

Sample preparation	Brains were harvested and tumour regions corresponding to tumour bulk, invaded striatum and corpus callosum were micro-dissected under fluorescent guidance in ice cold HEPES buffered HBSS. Micro-dissected regions were dissociated by incubating with papain (20 units/ml) DNase (0.005%) for 30min at 37°C (Worthington). Samples were triturated in HEPES buffered EBSS to fully dissociate the tissue and centrifuged (3 min, 300xg). Digestion was terminated by trituration in EBSS ovomucoid inhibitor (10mg/ml), albumin (10mg/ml) and DNase (0.005%). Following centrifugation (3 min, 300xg), cells were resuspended in 400µL FACs sorting buffer (1.5% BSA, 2.5mM HEPES, 1mM EDTA in PBS) containing 2.5% RNAsin and 1/10000 DAPI (Promega).<br>For isolation of O4+ tumour cells,cells were isolated as above and subjected to a further two centrifugation steps (1min at 200xg) in 10ml warm EBSS after digestion to reduce myelin debris. Following centrifugation in EBBS, cells were resuspended in 500ul GSC media containing 1:500 mouse anti-O4 (Alexa Fluor 594) and incubated for 15min at 37°C. Cells were washed once in PBS + 3% BSA and resuspended in 400µL FACs sorting buffer containing 1/10000 DAPI.
Instrument	Cell sorting was performed using the BD FACSAria III
Software	BD FACSDiva™ software was used for collection of data. FlowJo software was used to produce extended figure 4 exemplifying the gating strategy,.
Cell population abundance	Where possible, sorted samples were confirmed for purity post-sort via flow cytometry. Sorted populations were of >98% purity.
Gating strategy	All cells were first gated in FSC/SSC according to cell size and granularity. This population was then gated according to their viability using cell DAPI (negative population). Subsequently cells were gated according to GFP positivity (tumour cells) or negativity (non-tumour cells) for RNA-seq experiments. When sorting for subsequent live imaging experiments O4+ and O4- populations were further gated according to their positivity or negativity for surface marker O4.

☒ Tick this box to confirm that a figure exemplifying the gating strategy is provided in the Supplementary Information.
